# A transcriptomics-based computational drug repurposing pipeline identifies simvastatin and primaquine as therapeutics for endometriosis

**DOI:** 10.1016/j.isci.2026.117153

**Published:** 2026-08-21

**Authors:** Tomiko T. Oskotsky, Xinyu Tang, Erin Arthurs, Arpita Govil, Ferheen Abbasi, Arohee Bhoja, Daniel G. Bunis, Abby Lau, Jakob Einhaus, Maïgane Diop, Juan C. Irwin, Brice Gaudilliere, David K. Stevenson, Linda C. Giudice, Stacy L. McAllister, Marina Sirota

**Affiliations:** 1 Bakar Computational Health Sciences Institute, University of California, San Francisco, San Francisco, CA, USA; 2 Division of Clinical Informatics and Digital Transformation, Department of Medicine, University of California, San Francisco, San Francisco, CA, USA; 3 Department of Gynecology and Obstetrics, Emory University, Atlanta, GA, USA; 4 Center for Reproductive Sciences, Department of Obstetrics, Gynecology and Reproductive Sciences, University of California, San Francisco, San Francisco, CA, USA; 5 Department of Anesthesiology, Perioperative and Pain Medicine, Stanford University, Stanford, CA, USA; 6 Department of Pediatrics, Stanford University, Stanford, CA, USA; 7 Department of Pediatrics, University of California, San Francisco, San Francisco, CA, USA

**Keywords:** endometriosis, drug repurposing, transcriptomics, simvastatin, primaquine, vaginal hyperalgesia, RNA sequencing, gene expression reversal, real-world data, electronic medical records

## Abstract

Endometriosis has limited treatment options, prompting the search for data-driven therapeutics. We previously used a transcriptomics-based computational drug repositioning pipeline and identified several drug candidates. Fenoprofen, our top *in silico* candidate, was validated in a rat model of endometriosis-associated pain. Building on this, we evaluated two additional candidates, simvastatin and primaquine. Using the rat model, we conducted behavioral testing, bulk RNA sequencing, and differential expression analysis to assess their therapeutic potential. We also assessed endometriosis diagnosis among patients prescribed simvastatin in electronic medical records across six University of California (UC) healthcare institutions. Overall, simvastatin and primaquine attenuated pain-associated behaviors and reversed endometriosis-related gene expression changes in our animal model. Moreover, simvastatin prescription was associated with a lower observed relative risk of endometriosis in our retrospective multi-center cohort study. These findings highlight their potential as repurposed therapeutics for endometriosis and support the effectiveness of computational drug repositioning in identifying treatment strategies.

## Highlights

• A computational pipeline identified simvastatin and primaquine for endometriosis
• Simvastatin and primaquine reduced pain-related behavior in a rat endometriosis model
• Both drugs reversed endometriosis-linked gene expression changes in treated tissue
• Simvastatin use was associated with lower endometriosis risk in real-world patient records

## Graphical abstract

**Figure:**
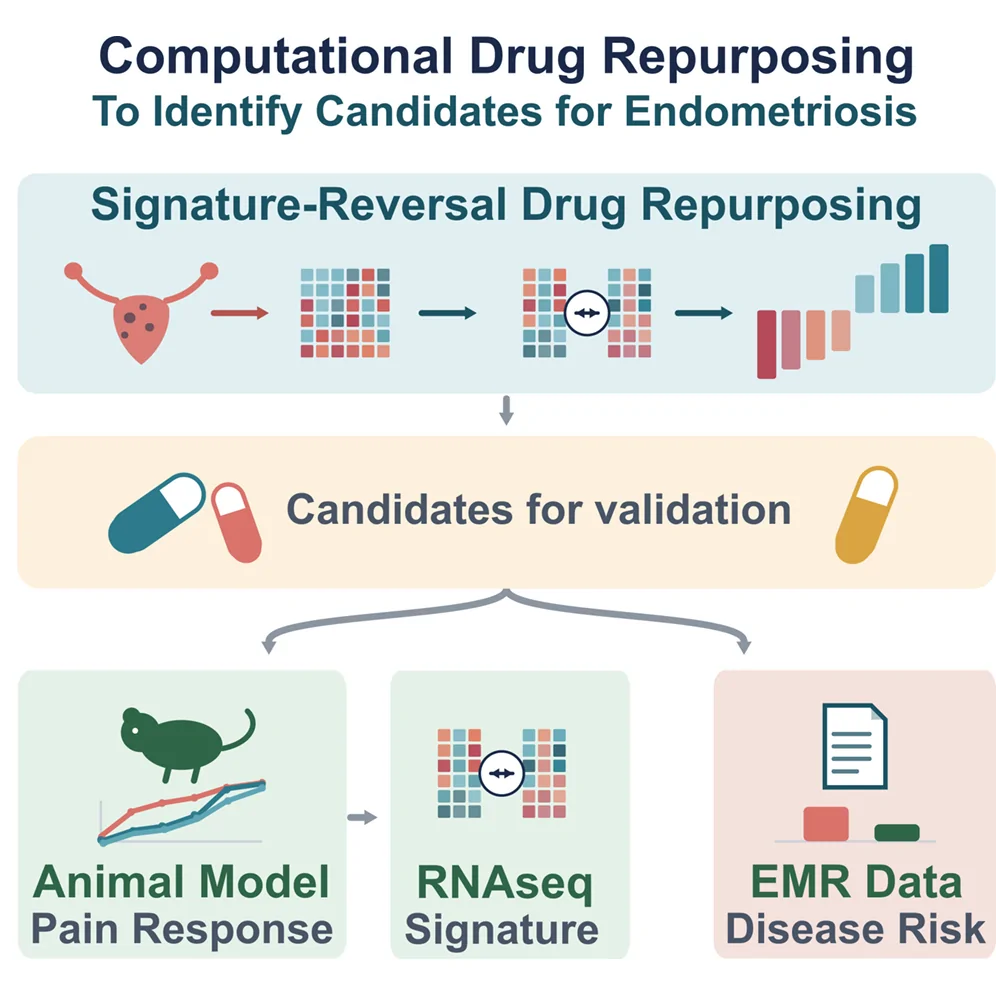

## Teaser

Health sciences; Medical specialty

## Introduction

Endometriosis is a common, life-altering disease, with symptoms including severe dysmenorrhea, chronic pelvic pain, and infertility.^1^ People with endometriosis can be undiagnosed for years, given non-specific symptoms that can overlap with other disorders and the requirement for surgical confirmation of disease.^1^ It has been suggested that 10% of reproductive-aged women have endometriosis,^2^ but this value may be an underestimate due to precise diagnostics requiring laparoscopy to identify and biopsy suspected endometriosis lesions. Approximately 12%–32% of menstruating women who have surgery for pelvic pain and 50% who have infertility have been found to have endometriosis.^3^ The disease is not only a significant public health issue, but it also has a large economic impact on health expenditures of almost $70 billion annually in the US.^4^

Endometriosis is characterized by growth of endometrial tissue outside the uterus that responds to cyclic hormonal changes, similar to uterine endometrial tissue during a normal menstrual cycle.^2^ These lesions commonly exist at extrauterine sites such as the pelvic peritoneum, ovaries, and bowel, where they elicit an inflammatory response, fibrosis, and pain.^5^ The eutopic endometrium in women with endometriosis also differs from that of those without endometriosis, showing aberrant nerve fiber infiltration, increased angiogenesis, and disrupted progesterone response.^6^^,^^7^ Although endometriosis was identified over 100 years ago, the disease remains with limited medical treatment options.^3^ Current evidence does not support any medication for primary prevention of endometriosis; however, as an estrogen-driven disease, hormonal therapies have been used to treat pain symptoms and reduce post-surgical recurrence.^2^^,^^8^^,^^9^^,^^10^ First-line treatments for dysmenorrhea and non-menstrual pelvic pain associated with endometriosis typically include estrogen-progestin and progestin-only contraceptives or gonadotropin-releasing hormone (GnRH) analogs, as well as nonsteroidal anti-inflammatory drugs (NSAIDs).^3^^,^^8^ Unfortunately, existing medical therapies for endometriosis-related pain are often ineffective, with individuals experiencing minimal or transient pain relief or intolerable side effects limiting long-term use.^5^ Surgical excision of lesions can relieve symptoms, although combining lesion excision with hysterectomy has been shown to yield greater improvements in pain and quality of life.^11^ Even after surgery, many patients continue to experience symptoms.^12^ This underscores the need for new endometriosis treatment strategies that provide effective, non-hormonal control of pain, minimize local and systemic inflammation associated with pain and infertility, and, where possible, reduce the risk of developing disease or recurrence after surgery. Development of new drugs for endometriosis has been challenging due to the heterogeneity among affected individuals, variable symptoms, and complex etiologies, as well as historically lower investment in women’s reproductive health conditions.^13^^,^^14^ Additionally, drug development for endometriosis has been limited as it is both costly and time-intensive, often requiring millions of dollars and decades of work, for therapeutics to finally become commercially available.^15^

Computational drug repositioning methods allow for identification of new therapeutic applications for drugs already on the market in a fraction of the time and funds that it takes to develop and test entirely new drugs.^16^ Our group developed a method that identifies potential therapeutics that reverse gene expression profiles of a disease, i.e., genes downregulated in the disease are upregulated by the drug, and vice versa.^17^ This approach leverages transcriptomics data to generate genome-wide expression profiles from comparisons between disease and healthy samples or drug-treated and untreated cells.^17^ In the past, this method has been successfully applied to identify both known and previously unrecognized treatments for inflammatory bowel disease,^18^ dermatomyositis,^19^ liver cancer,^20^ preterm birth,^21^ and Alzheimer’s disease.^22^ Our recent work to identify therapeutic candidates for endometriosis leveraged publicly available disease and drug gene-expression signatures in conjunction with a transcriptomics-based computational drug repositioning pipeline and validated the top candidate.^23^ Specifically, we applied this *in silico* approach to endometriosis disease signatures that were unstratified and stratified by American Society for Reproductive Medicine (ASRM) disease stage^24^ (I-II and III-IV) and by menstrual cycle phase (proliferative, early secretory, and mid secretory) and found 299 drugs that reversed the endometriosis disease signature.^23^ Our top candidate, fenoprofen, an uncommonly prescribed non-steroidal anti-inflammatory drug (NSAID) in the US, successfully alleviated vaginal hyperalgesia in a rat model of endometriosis.^23^

In the current study, we selected two therapeutic candidates from our earlier drug repositioning work for further investigation, the cholesterol-lowering drug simvastatin and the antimalarial agent primaquine, based on their strong reversal of the endometrial signature in cases versus controls and good safety profiles.^25^^,^^26^ Statins and anti-malarial agents have previously been considered potential therapeutics for endometriosis.^27^^,^^28^^,^^29^^,^^30^^,^^31^^,^^32^^,^^33^^,^^34^^,^^35^^,^^36^^,^^37^^,^^38^^,^^39^^,^^40^ Herein, we provide further evidence for simvastatin and primaquine as potential endometriosis therapeutics. Specifically, we conducted behavioral studies using an established rat model of endometriosis to assess the effect of treatment with simvastatin and primaquine on pain endpoints. Furthermore, we performed RNA sequencing on the uteri and lesions from these rats and analyzed differentially expressed genes (DEGs) for treated vs. untreated (control) tissues. We also conducted a retrospective multi-center cohort study using a database of clinical records representing approximately 9.8 million individuals and assessed the risk of endometriosis among women prescribed simvastatin compared to those who were not prescribed this drug.

## Results

### Overview

**Figure 1:**
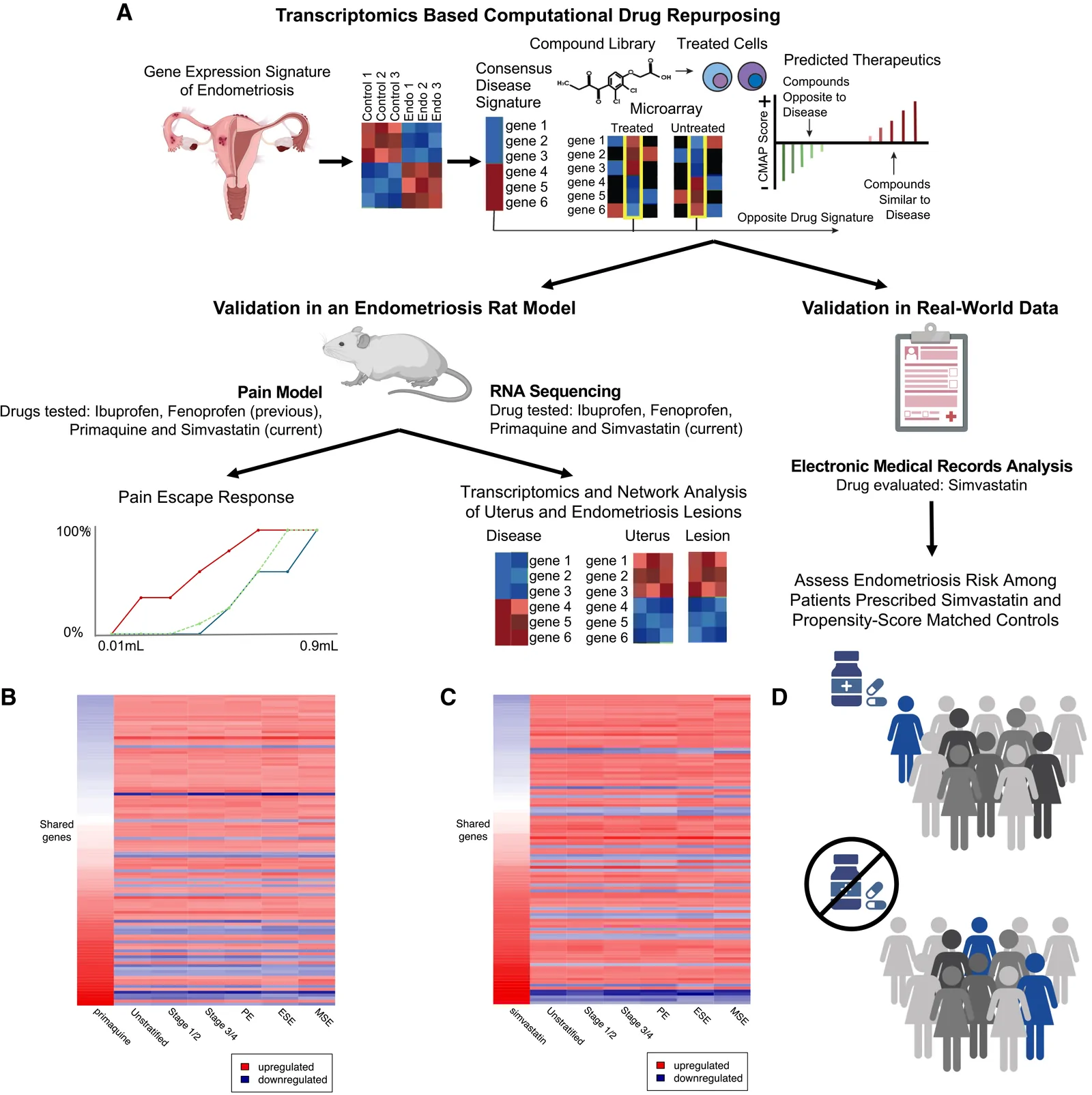
Study overview and disease signature reversal by primaquine and simvastatin (A) Overview of the study that identified therapeutic candidates through a transcriptomics-based computational drug repositioning pipeline and then validated drug candidates in a rat model of endometriosis and in real-world data. Bulk RNA-sequencing was conducted of the uterus and endometriosis lesions from the rats, and differentially expressed genes and enriched pathways were analyzed. (B and C) Heatmap showing (B) primaquine and (C) simvastatin drug signature (far-left column of heatmaps) vs. unstratified and stratified (by stage and menstrual cycle phase) endometriosis disease signatures (remaining columns of heatmaps). (D) Endometriosis risk was assessed among patients exposed to simvastatin and propensity-score matched controls through analysis of electronic medical records data.

An overview of our study is provided in Figure 1A. We previously leveraged a transcriptomics-based computational drug repositioning pipeline^17^ using gene expression signatures of endometriosis queried against the Connectivity Map (CMap) database,^41^ and identified fenoprofen as the top drug among 299 unique therapeutic candidates^23^ (Figure 1A and Table S1). Fenoprofen alleviated vaginal hyperalgesia comparably to our positive control, ibuprofen, in our rat endometriosis model.^23^ Among the compounds of interest with the greatest endometriosis disease-signature reversal, we selected two therapeutic candidates for further evaluation in this current study. In our endometriosis animal study model, we assessed pain-associated behavior in animals that were treated with each compound and compared them to untreated animals. We then performed RNA sequencing of the uteri and endometriosis lesions to assess the gene expression from these tissues in the treated and untreated animals.

### Computational drug repositioning pipeline identifies primaquine and simvastatin as therapeutic candidates for endometriosis

Among 299 drugs identified from the unstratified and ASRM disease stage- and cycle phase-stratified signatures, the cholesterol-lowering drug simvastatin and antimalarial agent primaquine were endometriosis therapeutic candidates selected for further study based on criteria including their strong reversal scores, safety profiles, and availability. When visualizing the gene expression of the six input endometriosis signatures (i.e., unstratified, and stratified by stage [I-II or III-IV] and phase [proliferative, early secretory, mid-secretory]) in comparison to the signature of primaquine from CMap (Figure 1B), the overall reversal pattern can be observed. A similar overall pattern of reversal can be seen when visualizing the gene expression of the six input endometriosis signatures and simvastatin (Figure 1C).

### Primaquine and simvastatin attenuate escape responses in a rat model of endometriosis

The effect of our candidate drugs on endometriosis-associated vaginal hyperalgesia, a surrogate marker for endometriosis-related pain, was assessed in a rat model of endometriosis.

#### Vehicle

**Figure 2:**
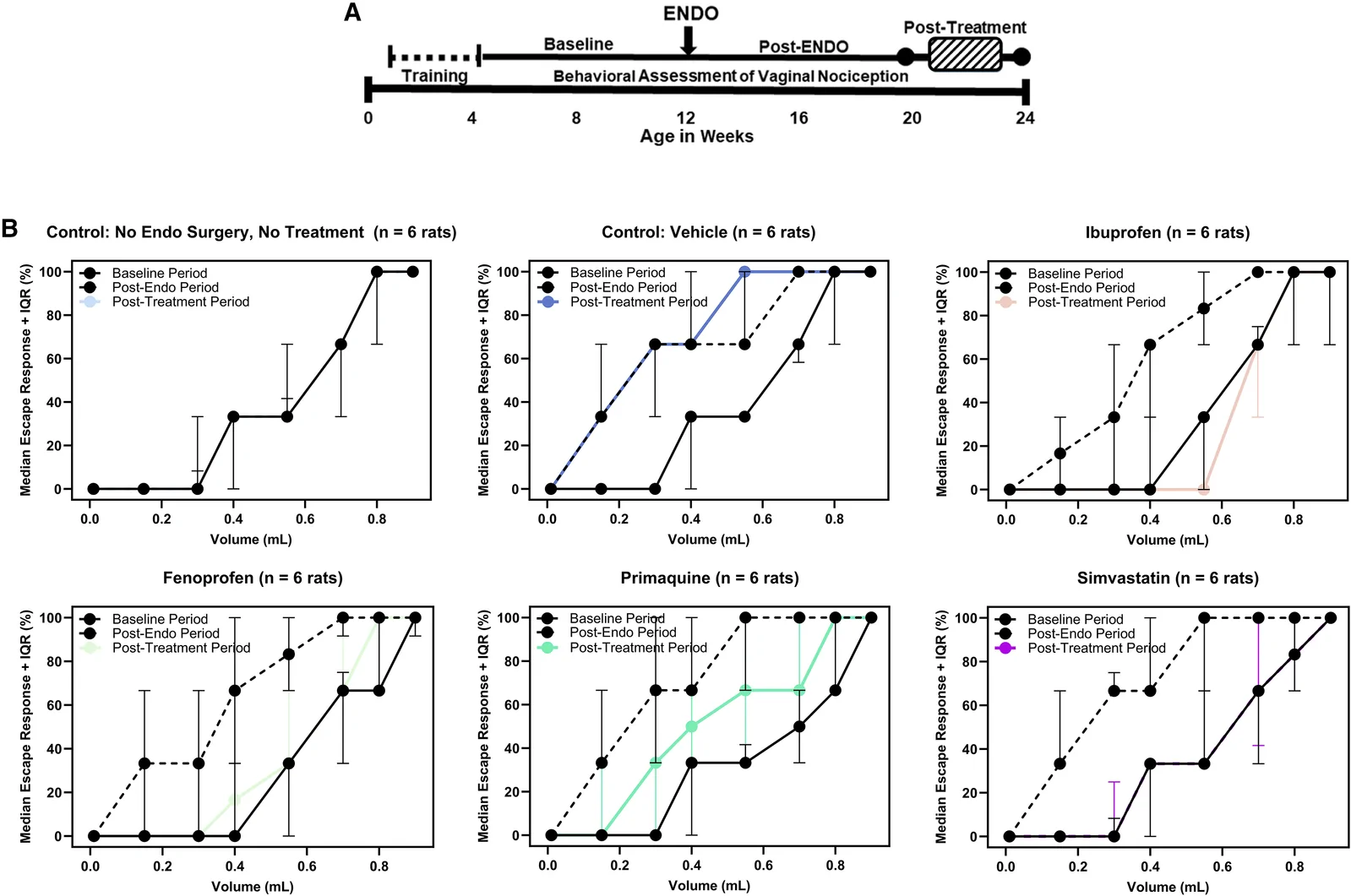
Endometriosis pain-associated escape behaviors in a rat model (A) Female Sprague-Dawley rats (*n* = 6 per group) were trained over 4 weeks to perform an escape response to terminate a noxious vaginal stimulus (water-filled balloon). Vaginal nociception was assessed as % escape response to varying balloon volumes in 1-h sessions, 3 times/week, over 24 weeks. Each session included 8 volumes (0.01–0.90 mL), each tested 3 times in randomized, blinded order. Responses to none of the trials were counted as 0% escape response, 1 out of 3 trials as 33% escape response, 2 out of 3 trials as 67% escape response, and all 3 trials of a volume were counted as 100% escape response. Nociception was measured at baseline (for 8 weeks), after endometriosis induction (post-endo, for 8 weeks), and during a 4-week treatment period. (B) Animal model median escape response (%) with interquartile range (IQR; error bars) (*y* axis) for each delivered volume (0.01, 0.15, 0.30, 0.40, 0.55, 0.70, 0.80, and 0.90 mL) (*x* axis) during the baseline, post-endo surgery, and post-treatment periods for the 6 animal study groups; control no endo surgery; vehicle; ibuprofen; fenoprofen; primaquine; and simvastatin. (Note: control no endo surgery, ibuprofen, and fenoprofen escape responses results are from previous work.^23^). Mann-Whitney U test; significance threshold of 0.05 applied to Bonferroni-correct *p* values. See Figure S1 for *p* values.

Among rats that received endometriosis (“endo”) surgery and vehicle treatment (*n* = 6), escape responses were significantly increased during the post-endo period compared to the baseline period, when volumes of 0.15, 0.30, 0.40, 0.55, 0.70, and 0.80 mL of water were delivered intra-vaginally (Mann-Whitney U test, Bonferroni-corrected *p* value threshold of 0.05. Figures 2B and S1). During the post-treatment period for the vehicle treatment group, similar to the post-endo period, escape responses were significantly increased relative to the baseline period when volumes of 0.15, 0.30, 0.40, 0.55, 0.70, and 0.80 mL of water were delivered (Mann-Whitney U test, Bonferroni-corrected *p* value threshold of 0.05. Figures 2B and S1). In the vehicle group, no statistically significant differences were found between the post-endo period and the post-treatment period escape responses for any volume of water delivered (Mann-Whitney U test, Bonferroni-corrected *p* value threshold of 0.05. Figures 2B and S1).

#### Primaquine

Among rats that received endo surgery and primaquine treatment (40 mg/kg/day, orally or “per os” [p.o.]) (*n* = 6), escape responses were significantly increased during the post-endo period compared to the baseline period, when volumes of 0.15, 0.30, 0.40, 0.55, 0.70, and 0.80 mL of water were delivered (Mann-Whitney U test, Bonferroni-corrected *p* value threshold of 0.05. Figures 2B and S1). During the post-treatment period, escape responses were significantly decreased compared to the post-endo period, when volumes of 0.15, 0.30, 0.40, 0.55, and 0.70 mL of water were delivered (Mann Whitney U test, Bonferroni-corrected *p* value threshold of 0.05. Figures 2B and S1). Relative to the baseline period, post-treatment escape responses were significantly increased, when volumes of 0.30, 0.40, 0.55, and 0.70 mL of water were delivered (Mann-Whitney U test, Bonferroni-corrected *p* value threshold of 0.05. Figures 2B and S1).

#### Simvastatin

Among rats that received endo surgery and simvastatin treatment (40 mg/kg/day, p.o.) (*n* = 6), escape responses were significantly increased during the post-endo period compared to the baseline period, when volumes of 0.15, 0.30, 0.40, 0.55, 0.70, and 0.80 mL of water were delivered (Mann-Whitney U test, Bonferroni-corrected *p* value threshold of 0.05. Figures 2B and S1). During the post-treatment period, escape responses were significantly decreased compared to the post-endo period when volumes of 0.15, 0.30, 0.40, 0.55, 0.70, and 0.80 mL of water were delivered (Mann-Whitney U test, Bonferroni-corrected *p* value threshold of 0.05. Figures 2B and S1). In the simvastatin group, no statistically significant differences were found between the baseline period and the post-treatment period escape responses for any volume of water delivered (Mann-Whitney U test, Bonferroni-corrected *p* value threshold of 0.05. Figures 2B and S1).

#### Comparison across groups

Overall, the pattern of escape responses for primaquine (“PRIMA”) and simvastatin (“SIMVA”) are similar to those previously shared for fenoprofen-treated (“FEN”) and ibuprofen-treated (“IBU”, positive control) rats^23^ (Figures 2B and S1).

**Figure 3:**
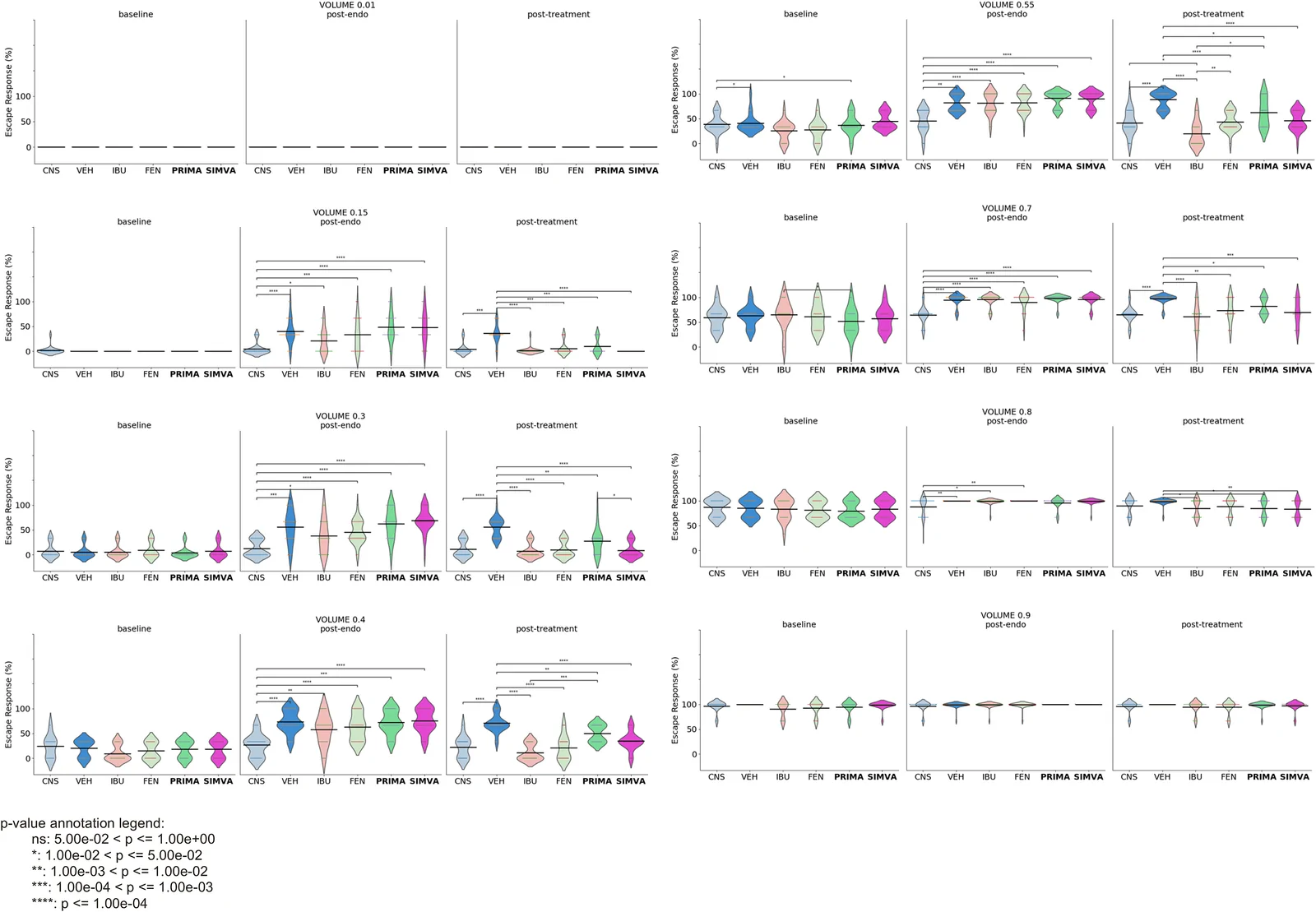
Strip and violin plots of escape responses Strip and violin plots with medians and significance bars of escape responses to vaginal balloon distension at volumes of 0.01, 0.15, 0.30, 0.40, 0.55, 0.70, 0.80, and 0.90 mL at baseline (baseline), post-endometriosis (post-endo), and post-treatment (post-treatment) periods in a rat endometriosis model comparing 6 animal study groups—control no surgery (“CNS”), vehicle (“VEH”), ibuprofen (“IBU”), fenoprofen (“FEN”), primaquine (“PRIMA”), simvastatin (“SIMVA”). Mann-Whitney U test; Bonferroni adjusted *p* values from Mann-Whitney U comparisons of groups. (Note: CNS, FEN, and IBU escape responses results are from previous work.^23^). See Table S2 for exact adjusted *p* values. *p* value annotation legend: ns: 5.00e−02 < *p* ≤ 1.00e+00. ∗: 1.00e−02 < *p* ≤ 5.00e−02. ∗∗: 1.00e−03 < *p* ≤ 1.00e−02. ∗∗∗: 1.00e−04 < *p* ≤ 1.00e−03. ∗∗∗∗: *p* ≤ 1.00e−04.

During the baseline period, there was a significant difference in escape responses between Control No endo Surgery (“CNS”) and Vehicle (“VEH”), and CNS and PRIMA at 0.55 mL, and between PRIMA and IBU at 0.7 mL, otherwise no other significant differences were identified between the 6 animal study groups (Figure 3 and Table S2).

During the post-endo period, there were significant differences between the negative control CNS and the 5 other groups at volumes 0.15, 0.3, 0.4, 0.55, and 0.7 mL; and between CNS and three other groups—VEH, IBU, and FEN—at 0.8 mL. No significant differences were identified between any of the six groups at 0.01 and 0.9 mL (Figure 3 and Table S2).

During the post-treatment period, there were significant differences in escape responses between the negative control VEH and the other 4 drug-treatment groups—IBU, FEN, PRIMA, and SIMVA—at volumes 0.15, 0.3, 0.4, 0.55, 0.7, and 0.8 mL; however, no significant differences between the groups were identified at 0.01 or 0.9 mL. There was a significant difference between CNS and IBU at 0.55 mL; otherwise, there were no significant differences in escape responses between CNS and the four drug-treatment groups—IBU, FEN, PRIMA, and SIMVA. Between the drug-treatment groups, there were significant differences in escape responses between PRIMA and SIMVA at 0.3 mL, PRIMA and positive control IBU at volumes 0.4 and 0.55 mL, and FEN and positive control IBU at volume 0.55 mL; otherwise no other significant differences were identified between positive control IBU, FEN, PRIMA, and SIMVA (Figure 3 and Table S2).

### RNA sequencing analysis of treated animals confirms reversal of disease signatures

To assess the impact of our candidate drugs on gene expression, bulk RNA sequencing was performed on the uterus and endometriosis-like “lesions” from rats (*n* = 6 per group per sample source).

#### Principal component analysis

**Figure 4:**
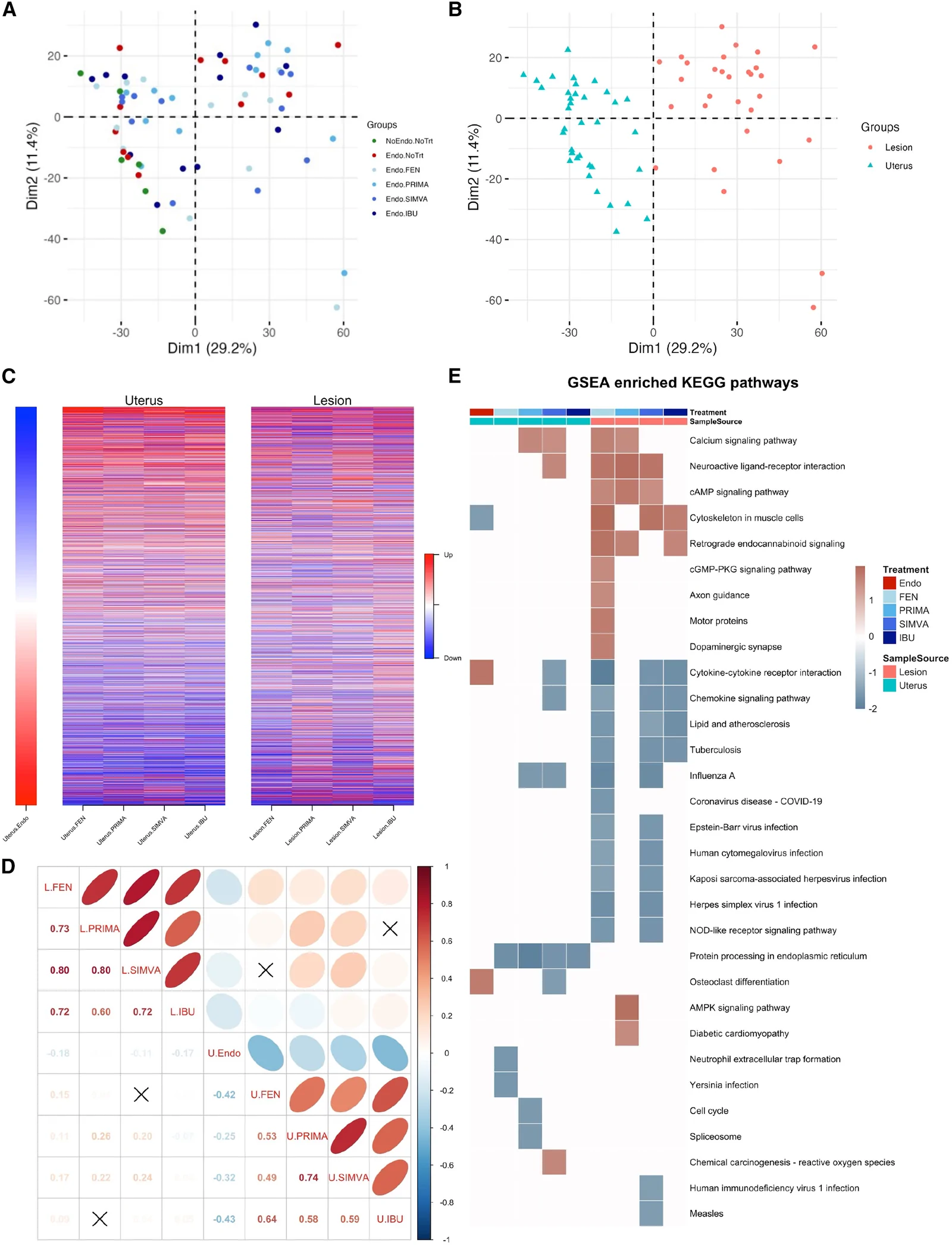
RNA-Seq analysis of treated animals Principal component analysis (PCA) of all animal study samples, with each dot representing a sample, colored (A) by treatment group or (B) by sample source. (C) Heatmap of disease-associated (i.e., DEGs of endometriosis vs. control) and treatment-associated (i.e., DEGs of treated vs. control) gene signatures. (Left: vehicle-treated uterus samples. Middle: drug-treated uterus samples. Right: lesion samples). Red indicates up-regulated genes and blue indicates downregulated genes. (D) Pearson correlation analysis between disease-induced (“U.Endo”) and drug-induced gene expression changes. Red indicates positive correlations and blue indicates negative correlations. (E) Gene set enrichment analysis (GSEA) of KEGG pathways in disease and drug-treated groups. Colors represent the GSEA normalized enrichment score (NES) to compare the directionality of pathway responses across groups. Red indicates up-regulated pathways and blue indicates downregulated pathways. See related Figures S2–S4 and Tables S3, S4, S5, S6, S7, S8, S9, S10, and S11.

Principal component analysis (PCA) revealed that samples from different treatment groups did not show distinct clustering (Figure 4A). Instead, the sample source (i.e., uterus or lesion) was the primary factor contributing to the differences between samples (Figure 4B). PCA was subsequently conducted within each sample source which still did not show distinct clustering based on treatment groups (Figure S2).

#### Differential gene expression

Differential gene expression analysis of the lesion and uterus samples between endometriosis rats with and without treatment revealed varying numbers of DEGs (Figures 4, S3 and S4 and Tables S3, S4, S5, S6, S7, S8, S9, S10, and S11). Comparison of uterine samples from control rats and vehicle-treated rats that underwent endometriosis surgery revealed gene expression patterns associated with the disease (Figure 4C, left). Further comparisons of the disease-associated gene expression patterns with those from drug-treated rats demonstrated that the four drugs—primaquine and simvastatin, as well as fenoprofen and positive control ibuprofen—effectively reversed the gene expression profile in the uterus (Figure 4C, middle), with significant negative correlations (Figure 4D, bottom right). In the treated lesions, reversal of the gene expression profile associated with the disease was observed, particularly with fenoprofen, simvastatin, and ibuprofen (Figure 4C, right), although compared with the treated uterine samples (Figure 4C, middle), this reversal in the treated lesions appears less pronounced (Figure 4C, right), and with only weak negative correlations (Figure 4D, top left).

#### Gene set enrichment analysis

From gene set enrichment analysis, pathways enriched in the uterus before and after drug treatment were identified. *Cytokine-cytokine receptor interaction* and *osteoclast differentiation* were upregulated KEGG (Kyoto Encyclopedia of Genes and Genomes) pathways in the uteri of endometriosis rats without drug treatment (i.e., vehicle), while *cytoskeleton in muscle cells* was a downregulated KEGG pathway. Simvastatin was able to reverse the *cytokine-cytokine receptor interaction* and *osteoclast differentiation* pathways in the uterus. Additionally, fenoprofen, simvastatin, and ibuprofen were able to reverse the *cytokine-cytokine receptor interaction* and *cytoskeleton in muscle cell pathways* in the lesion, although these effects were not strongly reflected at the overall gene expression level (Figure 4E).

#### Network analysis

**Figure 5:**
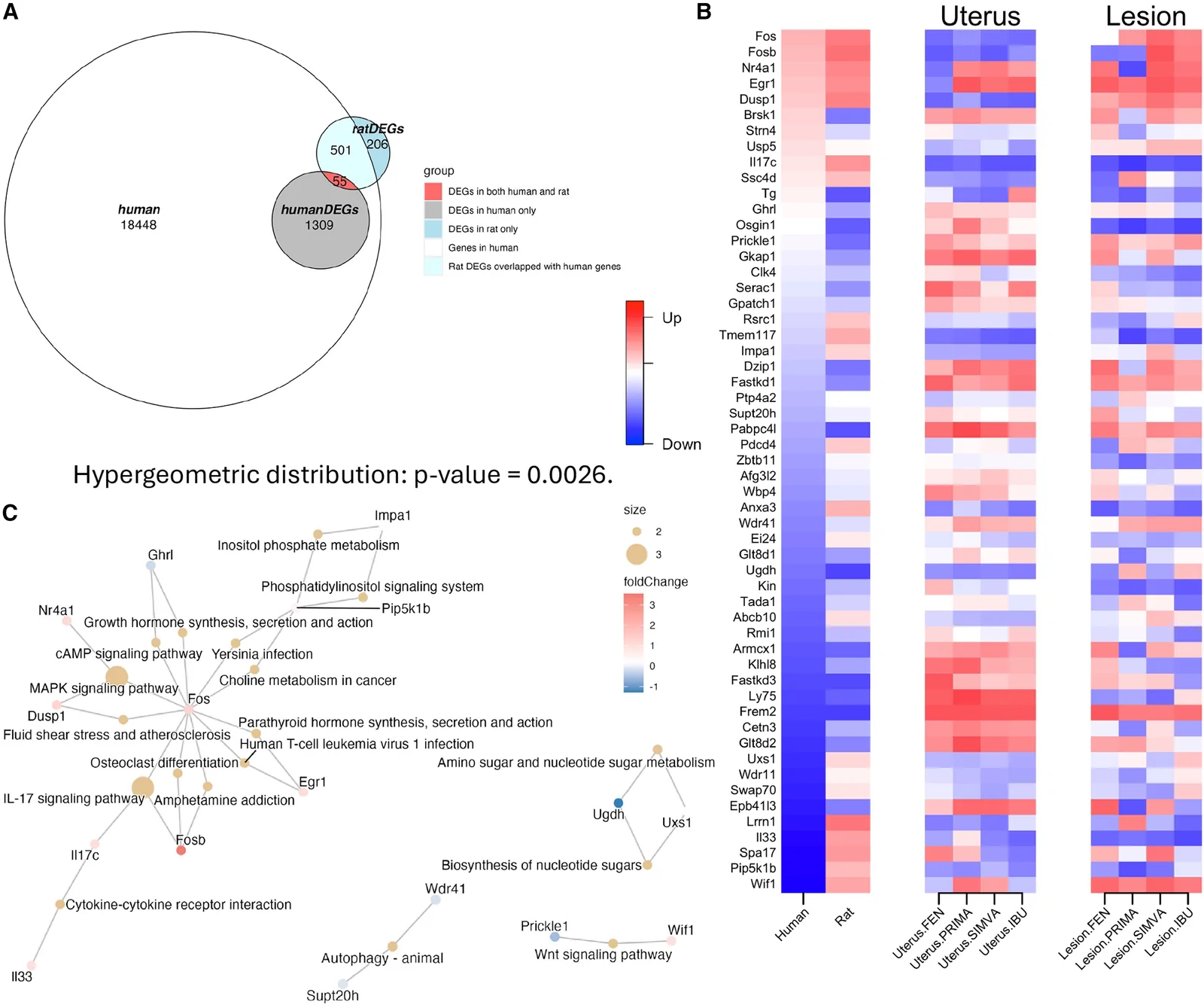
Correlation with human data (A) Venn diagram demonstrating overlap of rat and human endometriosis signatures, where the overlap between human and rat DEGs was evaluated using the hypergeometric test. (B) Heatmap reflecting the expression profiles of the 55 overlapping DEGs in humans, and in rats in the uterus and the lesion samples with and without drug treatment. (C) Network analysis to identify pathways associated with the 55 DEGs that overlap between our human and rat transcriptomics analysis, and their networks. The rat disease signature was defined using a nominal *p* value <0.05 cutoff. The human disease signature was defined using cutoffs of adjusted *p* value <0.05.

To explore the similarities between the rat model and humans, the rat endometriosis gene signatures were compared to the human signatures reported in previous work,^23^^,^^42^ resulting in the identification of 55 overlapping DEGs (Figure 5A). A hypergeometric test on rat DEGs with human homologs confirmed their overrepresentation in human signatures. The expression profiles of these overlapping genes in the uterus and the lesion samples with and without drug treatment are visualized in Figure 5B. Despite the foreseeable discrepancies in the expression profiles in humans and rats, several genes, such as Fos proto-oncogene (human *FOS*, rat *Fos)*, FosB proto-oncogene (human *FOSB*, rat Fosb*)*, interleukin 17C (human *IL17C*, rat *Il17c)*, nuclear receptor subfamily 4 group A member 1 (human *NR4A1*, rat *Nr4a1)*, dual specificity phosphatase 1 (human *DUSP1*, rat *Dusp1)*, lymphocyte antigen 75 (human *LY75* or *DEC-205*, rat *Ly75*), and FRAS1-related extracellular matrix 2 (human *FREM2*, rat *Frem2)* showed consistency across species. *FOS* and *FOSB*, members of the Fos gene family, encode Fos proteins which have previously been associated with endometriosis.^43^^,^^44^ *NR4A1*^45^^,^^46^ and *LY75*^47^ have also been linked with endometriosis, whereas *IL17C*, *DUSP1*, and *FREM2* are less well-characterized in the context of this condition. Notably, the four drugs reversed or partially reversed the expression of these genes in the uterus of endometriosis rats (Figure 5B). To identify pathways associated with these 55 genes, we conducted a network analysis, revealing *cytokine-cytokine receptor interaction*, *IL-17 (interleukin-17) signaling*, and *MAPK (mitogen-activated protein kinase) signaling* as the primary pathways involved (Figure 5C).

### Simvastatin prescription is associated with fewer endometriosis diagnoses in electronic medical records analysis

Among approximately 9.8 million patients across six University of California health centers (January 1, 2012–May 30, 2025) who were represented in the deidentified limited electronic medical records (EMRs) dataset, 732 individuals had a prescription for primaquine and 265,754 individuals had a simvastatin prescription. After applying inclusion and exclusion criteria, the compound of interest (COI)-prescribed cohorts comprised 68 patients for primaquine (too few for statistically meaningful analysis) and 2,336 patients for simvastatin (Figures 1 and S5).

Cohort characteristics and standardized mean differences (SMDs) of covariates between simvastatin-prescribed and control groups showed adequate balance after propensity score matching by demographics (age, race, ethnicity), UC healthcare site, number of visits, and medication indication (high cholesterol/hyperlipidemia), with the absolute value of SMD of less than 0.1 for all covariates after matching (Figure S6 and Tables 1 and 2).

**Table 1:** Cohort demographic characteristics before propensity score matching with absolute SMDs for patients prescribed simvastatin compared with control patients not treated with simvastatin

Characteristic	None	Simvastatin	SMD
*n*	2,853,040	2,336	–
Year of Birth (mean [SD])	1974.95 (20.80)	1981.68 (6.52)	0.436
Age (median [IQR])	48.00 [33.00, 66.00]	44.00 [40.00, 48.00]	0.43
Race (%)	-	-	0.225
American Indian or Alaska Native (%)	15,203 (0.5)	21 (0.9)	–
Asian (%)	375,083 (13.1)	263 (11.3)	–
Black or African American (%)	177,701 (6.2)	204 (8.7)	–
Native Hawaiian or Other Pacific Islander (%)	18,412 (0.6)	28 (1.2)	–
Other Race (%)	546,376 (19.2)	608 (26.0)	–
White (%)	1,720,265 (60.3)	1,212 (51.9)	–
Ethnicity Hispanic or Latino (%)	603,163 (21.1)	782 (33.5)	0.28
Location (%)	-	-	0.331
UC1 (%)	451,026 (15.8)	306 (13.1)	–
UC2 (%)	534,668 (18.7)	631 (27.0)	–
UC3 (%)	361,474 (12.7)	470 (20.1)	–
UC4 (%)	674,491 (23.6)	413 (17.7)	–
UC5 (%)	817,636 (28.7)	502 (21.5)	–
UC6 (%)	13,745 (0.5)	14 (0.6)	–
Number of visits (mean [SD])	31.82 (71.36)	85.39 (140.29)	0.481
High cholesterol/hyperlipidemia (%)	388,412 (13.6)	1,189 (50.9)	0.87
Endometriosis (%)	30,944 (1.1)	67 (2.9)	0.128

**Table 2:** Cohort demographic characteristics after propensity score matching with absolute SMDs for patients prescribed simvastatin compared with control patients not treated with simvastatin

Characteristic	None	Simvastatin	SMD
*n*	9,344	2,336	–
Year of Birth (mean (SD))	1981.67 (6.60)	1981.68 (6.52)	0.001
Age (median [IQR])	44.00 [40.00, 48.00]	44.00 [40.00, 48.00]	<0.001
Race (%)	–	–	0.041
American Indian or Alaska Native (%)	101 (1.1)	21 (0.9)	–
Asian (%)	988 (10.6)	263 (11.3)	–
Black or African American (%)	774 (8.3)	204 (8.7)	–
Native Hawaiian or Other Pacific Islander (%)	132 (1.4)	28 (1.2)	–
Other Race (%)	2,519 (27.0)	608 (26.0)	–
White (%)	4,830 (51.7)	1,212 (51.9)	–
Ethnicity Hispanic or Latino (%)	3,251 (34.8)	782 (33.5)	0.028
Location (%)	–	–	0.095
UC1 (%)	1,264 (13.5)	306 (13.1)	–
UC2 (%)	2,746 (29.4)	631 (27.0)	–
UC3 (%)	1,785 (19.1)	470 (20.1)	–
UC4 (%)	1,374 (14.7)	413 (17.7)	–
UC5 (%)	2,104 (22.5)	502 (21.5)	–
UC6 (%)	71 (0.8)	14 (0.6)	–
Number of visits (mean (SD))	73.27 (127.14)	85.39 (140.29)	0.091
High cholesterol/hyperlipidemia (%)	4,757 (50.9)	1,189 (50.9)	<0.001
Endometriosis (%)	357 (3.8)	67 (2.9)	0.053

The rate of endometriosis among simvastatin-prescribed patients was 2.9% (67 of 2,336) and among matched untreated control patients ranged from 3.8% (357 of 9,344) to 4.1% (385 of 9344), corresponding to a lower observed relative risk (RR) of endometriosis diagnosis among simvastatin-prescribed patients (0.75 [95% confidence interval (CI), 0.58–0.97]; Benjamini-Hochberg (BH)-corrected *p* value = 0.03, with any reduction in the observed RR significant for ten of ten iterations) (Table 3).

**Table 3:** Relative risk of endometriosis in simvastatin-prescribed patients versus propensity score-matched unexposed controls (matched on age, race/ethnicity, UC health center, high cholesterol/hyperlipidemia, and number of visits)

Iteration	Exposed to simvastatin, endometriosis, *n* (%), (*n* total = 2,336)	Matched controls, endometriosis, *n* (%), (*n* total = 9,344)	RR [95% CI]	*p* value (BH-corrected)
1	67 (2.9)	375 (4.0)	0.71 [0.55–0.92]	0.018
2	67 (2.9)	385 (4.1)	0.70 [0.54–0.90]	0.018
3	67 (2.9)	365 (3.9)	0.73 [0.57–0.95]	0.021
4	67 (2.9)	375 (4.0)	0.71 [0.55–0.92]	0.018
5	67 (2.9)	357 (3.8)	0.75 [0.58–0.97]	0.03
6	67 (2.9)	374 (4.0)	0.72 [0.55–0.93]	0.018
7	67 (2.9)	373 (4.0)	0.72 [0.56–0.93]	0.022
8	67 (2.9)	362 (3.9)	0.74 [0.57–0.96]	0.022
9	67 (2.9)	378 (4.0)	0.71 [0.55–0.92]	0.022
10	67 (2.9)	369 (3.9)	0.73 [0.56–0.94]	0.018

Relative risk (RR) with 95% confidence intervals (95% CI) and Benjamini-Hochberg (BH)-corrected *p* values are reported for each iteration.

## Discussion

Effective therapeutics for endometriosis are in great need; however, developing new drugs for endometriosis is challenging due to the disease’s heterogeneity and complex symptoms, and the requirement of substantial resources and protracted timelines to bring new treatments to market.^13^^,^^15^ Bioinformatics approaches can help identify existing drugs that can be repurposed to treat this condition.^16^ Our work leveraging publicly available endometrial disease and drug gene-expression signatures and a transcriptomics-based computational drug repositioning pipeline identified several Food and Drug Administration (FDA)-approved drugs, including simvastatin and primaquine.^23^ While statins have been considered for some time as a potential treatment for endometriosis,^27^^,^^28^^,^^29^^,^^30^^,^^31^^,^^32^^,^^33^^,^^34^^,^^35^^,^^36^^,^^37^^,^^38^^,^^48^^,^^49^ anti-malarial agents have received comparatively less attention in this regard.^39^^,^^40^ *In vitro* studies evaluating the effect of simvastatin on human endometrial stromal cell cultures have found that simvastatin inhibits cell growth in a concentration-dependent manner,^29^^,^^30^^,^^31^ and that lipid-soluble statins, including simvastatin, decrease stromal cell invasiveness.^32^^,^^33^ A small prospective, randomized, double-blinded, controlled clinical trial (*n* = 60) comparing treatment with simvastatin versus a GnRH agonist in women who underwent laparoscopic surgery for pelvic endometriosis found significant reductions in dyspareunia, dysmenorrhea, and pelvic pain within both treatment groups over the 6-month postoperative period.^34^ No statistically significant differences were observed between the two treatment groups.^34^ A study of hydroxychloroquine found that treatment with this anti-malarial, *in vitro*, reduced endometrial and endometriotic cell survival and decreased the number of lesions in a mouse endometriosis model.^39^ Another study of the anti-malarial chloroquine and MK2206, an inhibitor of the serine/threonine protein kinase Akt (protein kinase B), found that combination therapy more effectively inhibited deep endometriotic stromal cell growth and reduced implant size in a mouse endometriosis model than either drug alone.^40^

Our work leverages a rodent endometriosis model, RNA sequencing, and real-world data to assess simvastatin and primaquine as potential therapeutics for endometriosis that could alleviate endometriosis-related pain with retrospective human data providing supportive evidence of an association between simvastatin prescription and fewer endometriosis diagnoses. While clinical records across the six healthcare centers included too few primaquine-prescribed patients for meaningful analysis, the dataset contained thousands prescribed simvastatin. Among 2,336 women who were prescribed simvastatin at age 40 or younger and had no prior diagnosis of endometriosis, we observed a statistically significant lower observed relative risk of endometriosis diagnosis compared with 9,344 propensity-score-matched controls who were never prescribed simvastatin. The findings from our analysis of real-world data are consistent with an association between simvastatin prescription and fewer endometriosis diagnoses.

We tested primaquine and simvastatin in a rat model of endometriosis, and found that treatment with each drug significantly alleviated vaginal hyperalgesia, a surrogate marker for endometriosis-related pain. Our findings with simvastatin were comparable to our prior findings with fenoprofen, the top drug candidate from our drug repositioning work, and with the positive control ibuprofen.^23^ There was a significant decrease in escape responses in the post-treatment period compared to the post-endo period (i.e., the period after the surgical induction of endometriosis). Furthermore, the degree to which simvastatin treatment attenuated the escape response was essentially the same as treatment with fenoprofen or ibuprofen, where there were no statistically significant differences between the baseline period and the post-treatment period.^23^ We found, however, that primaquine appeared less effective than ibuprofen, fenoprofen, and simvastatin at attenuating the escape response. In endometriosis rats with no treatment, vaginal hyperalgesia was maintained, confirming that the reduction in hyperalgesia seen in the treatment groups was not a result of additional vaginal nociceptive testing following the establishment of endometriosis. Overall, the findings from our current animal study work support our drug candidates, especially simvastatin, as therapeutics that could be used for endometriosis-related pain.

Comparative analysis of gene expression in our animal model showed that treatment with primaquine, simvastatin, fenoprofen, and ibuprofen effectively reversed the disease-associated profile in the uteri, with significant negative correlations, which could reflect how the drugs are affecting aberrances within the uterus that are associated with endometriosis. Treatment with these drugs induced more modest yet directionally similar changes in the lesions, suggesting transcriptional responses to treatment that vary in degree by site. A plausible explanation for the limited transcriptomic reversal within lesions is that systemically administered drugs achieve lower and less sustained pharmacodynamic exposure in the ectopic implants than in eutopic uterus. In surgically induced rat endometriosis, implanted tissue fragments can develop hypoxia,^50^ inflammation,^51^ and fibrotic encapsulation,^52^ conditions that could substantially restrict penetration of medications including the compounds tested here. Therapeutic efficacy may depend on achieving sufficient exposure in lesions. Consequently, surgically induced lesions may require longer treatment, higher dosing, localized delivery, or agents that directly target stromal remodeling or fibrosis to produce stronger transcriptional effects.

Among 55 endometriosis disease-associated genes that were found in common between humans and the rat model, several including *Fos*, *Fosb*, *Nr4a1*, and *Ly75*, showed consistency in the direction of their expression across species, signaling the critical role of these genes in disease pathology, and a pattern of reversal of expression after treatment by one or more of our drugs. *FOS* and *FOSB* are members of the Fos gene family, which encode Fos proteins, components of the activator protein-1 (AP-1) transcription factor complex that regulates key cellular processes such as proliferation, migration, and transformation. *FOS* gene and Fos protein expression were found to be higher in endometriotic tissue and eutopic endometrium in women with endometriosis relative to the eutopic endometrium of patients without endometriosis.^44^ *FOS* has also been found to be upregulated during endometriosis establishment in a baboon endometriosis model.^43^ NR4A1 has been found to be overexpressed in endometriosis and NR4A1 antagonists inhibit the growth of endometriotic lesions.^45^^,^^46^ Expression of *LY75*, involved in immune and inflammatory responses, has been found to be higher in patients with endometriosis compared to healthy control patients.^47^

Gene set enrichment analysis identified pathways affected in our endometriosis animal model, including *cytokine-cytokine receptor interaction* and *osteoclast differentiation* which were upregulated in the uterus, and *cytoskeleton in muscle cells* which was downregulated, and that simvastatin reversed the *cytokine-cytokine receptor interaction* and *osteoclast differentiation* pathways in the uterus, and fenoprofen, simvastatin, and ibuprofen reversed the *cytokine-cytokine receptor interaction* and *cytoskeleton in muscle cell* pathways in the lesions. Furthermore, network analysis identified pathways associated with the endometriosis disease-associated genes common across the two species, revealing *cytokine-cytokine receptor interaction*, *IL-17 signaling*, and *MAPK signaling* as primary pathways involved. *Cytokine-cytokine receptor interaction* and *IL-17 signaling* play important roles in host immunity and inflammation, and have been considered among targets for immune-mediated inflammatory disorders such as rheumatoid arthritis and adenomyosis.^53^^,^^54^^,^^55^ *MAPK signaling* is crucial in regulating cellular processes such as proliferation, differentiation, development, transformation, and apoptosis.^56^ Interestingly, these pathways have also previously been identified as enriched in studies of ectopic endometrium surrounding ovarian cysts compared to eutopic endometrium of patients with endometriosis (*cytokine-cytokine receptor interaction*, *IL-17 signaling*, and *MAPK signaling*)^57^ and human endometrial endothelial cells derived from eutopic endometrium of patients with and without endometriosis (*cytokine-cytokine receptor interaction*).^58^ The enrichment of these pathways, particularly among conserved DEGs, suggests their critical role in endometriosis. Moreover, these pathways may indicate the mechanistic targets of candidate drugs. Endometriosis is characterized by the growth of endometrial tissue outside of the uterus and local and systemic inflammation; thus, targeting these pathways could provide therapeutic strategies to manage the disease and alleviate its symptoms.

Our work demonstrates that simvastatin and primaquine, therapeutic candidates identified by our transcriptomics-based drug repositioning computational pipeline, effectively alleviate pain-associated behavior in an animal model of endometriosis. Additionally, these drugs successfully reversed the disease signature at the gene expression level in this animal model. Simvastatin, in particular, shows promise as a potential therapeutic for endometriosis-related pain. Moreover, our analysis of real-world data indicated that women prescribed simvastatin experienced fewer endometriosis diagnoses than matched unexposed patients, supporting an association that warrants evaluation in prospective clinical studies. The overlapping genes and pathways between our animal model and endometriosis patients offer insights into the biological processes associated with the disease and potential therapeutic targets. Also, our findings highlight the value of computational approaches in identifying therapeutic strategies for endometriosis.

### Limitations of the study

Our study has several strengths, including robust and well-validated analgesic effects demonstrated *in vivo*, cross-species molecular analyses supporting reversal of key inflammatory pathways, and validation using real-world clinical data; however, it also has several limitations. Transcriptomics from endometrium, a tissue accessible from those with and without endometriosis, rather than endometriosis lesions was used to identify drug repositioning candidates. Matched control tissue for such lesions was not available in this study, and we are unable to generate robust differential expression signatures from rat lesion tissue alone to identify a disease signature. Eutopic and ectopic endometria of women with endometriosis have been found to exhibit shared molecular alterations that are absent in the eutopic endometria of women without the disease.^59^ Eutopic endometrium can offer important insights into disease mechanisms^60^ and can serve as a practical foundation for therapeutic target discovery. Nevertheless, future work including signatures of the lesions themselves to query the drug data for therapeutic discovery would be valuable. Drugs not represented in the CMap dataset (e.g., ibuprofen and GnRH antagonists) would be missed by the drug repurposing pipeline. Similarly, drugs present in CMap but excluded during preprocessing due to inconsistent expression profiles also would not be identified.^20^ The drug repurposing pipeline focuses on identifying drugs that significantly reverse the overall endometriosis disease transcriptomic signature. This method does not necessarily consider whether the transcriptional effects of the drug are confined to the genes impacted by the disease. Therefore, a drug that induces extensive gene changes, including reversing the gene alterations caused by endometriosis, might be highlighted as a potential therapeutic option; however, such widespread gene changes may lead to unwanted side effects unrelated to the disease, which could limit the clinical usefulness of these drugs depending on the specificity and severity of the side effects and possible adverse pregnancy safety categories. Moreover, reversal of disease signatures by our drug candidates was assessed only in the uteri, and effects on fertility were not directly evaluated; however, all rats continued to exhibit normal estrous cycles throughout the study, suggesting that gross reproductive function was not disrupted. While we and others have extensively utilized the CMap dataset for therapeutic discovery in various non-cancer conditions,^18^^,^^19^^,^^21^^,^^22^^,^^23^ it is important to note that the compounds in the CMap dataset were tested on cancer cell lines. The effects of these drugs on endometrial tissue and disease lesions would provide a more accurate assessment of their potential applications for treating endometriosis. Unfortunately, a large number of these compounds has not yet been tested on relevant endometrial tissues. However, as new datasets become available, we will incorporate them into our future drug discovery efforts for endometriosis. Our bioinformatic findings are hypothesis-generating and, to strengthen them, would require experimental validation in future studies. A further limitation is that our candidate therapeutics were evaluated in a rodent model. While this model reproduces several features of human endometriosis, rats neither menstruate nor develop the disease spontaneously. Menstruating nonhuman primates develop endometriosis naturally and therefore represent a more suitable translational model; however, their close genetic relatedness to humans entails distinct ethical and regulatory challenges.^36^^,^^43^^,^^61^ Although prior work using this rat model showed that shamENDO does not produce vaginal hyperalgesia,^62^ the current study did not include concurrent shamENDO animals treated with vehicle, simvastatin, or primaquine. Therefore, we cannot directly determine whether either candidate drug alters baseline or shamENDO nociception or gene expression independent of the presence of endometriosis-like lesions. Prior work using this model has also shown that vaginal hyperalgesia does not necessarily correlate with lesion size,^62^^,^^63^ consistent with clinical observations that pain severity in endometriosis does not reliably track lesion burden.^2^^,^^10^ Accordingly, the current study was designed primarily to assess treatment effects on pain-associated behavior and lesion transcriptomic profiles rather than lesion burden. Although ectopic lesions were collected at endpoint for downstream RNA-seq analysis, lesion burden was not a preplanned endpoint, and lesion size and number were not prospectively recorded using a predefined, standardized analysis plan suitable for statistical comparison across treatment groups or correlation with behavioral or transcriptomic outcomes. Because treatment was initiated after endometriosis-like lesions were established and stabilized, this study was not designed to assess initial lesion development or determine whether simvastatin or primaquine altered lesion burden during treatment. Additionally, we evaluated a single dose level for both simvastatin and primaquine without formal pharmacokinetic (PK) or pharmacodynamic (PD) optimization or a dose response, which limits inferences about minimal effective doses and exposure response relationships. Findings from our electronic medical records analysis should be interpreted in light of limitations inherent to this type of data. Missing, incomplete, or inconsistently documented clinical information may have led to underascertainment or misclassification, particularly for care received outside the health system or information captured only in unstructured notes. These limitations may have affected observed associations. Also, our electronic medical records analysis was retrospective and therefore can establish association, not causation, between simvastatin exposure and lower observed frequency of endometriosis diagnosis. Medication histories and comorbidity data may be incomplete for some individuals, which could affect these findings. Prospective clinical trials are needed to determine therapeutic efficacy in endometriosis.

## Resource availability

### Lead contact

Further information and requests for resources and reagents should be directed to and will be fulfilled by the lead contact, Marina Sirota (marina.sirota@ucsf.edu).

### Materials availability

This study did not generate new unique reagents.

### Data and code availability

• Data that support the findings of this study were obtained from the University of California Data Discovery Platform (UCDDP), a HIPAA limited dataset which contains patient data from all six UC Health academic medical centers (Davis, Irvine, Los Angeles, Riverside, San Diego, and San Francisco). Due to privacy and confidentiality restrictions, the data are not publicly available. Minimal de-identified aggregate data in the form of tables are available from the corresponding author on reasonable request and subject to corresponding author’s institutional approval. UC researchers may obtain access after completing an initial analysis within their campus health system and securing approval for UC-wide extension. The University of California, San Francisco (UCSF) EMR database is available to UCSF-affiliated individuals who can contact UCSF’s Clinical and Translational Science Institute (CTSI) (ctsi@ucsf.edu) or the UCSF’s Information Commons team for more information (info.commons@ucsf.edu). University of California Data Discovery Platform (UCDDP) is only available to UC researchers who have completed analyses in their respective UC first and have provided justification for scaling their analyses across UC health centers. Additional instructions are available through the UC Health Center for Data-Driven Insights and Innovation (https://www.ucop.edu/uc-health/departments/center-for-data-driven-insights-and-innovations-cdi2.html). The animal study RNA seq data are available through the Gene Expression Omnibus (GEO), accession ID GEO: GSE296883 (https://www.ncbi.nlm.nih.gov/geo/query/acc.cgi?acc=GSE296883).
• Code for transcriptomic data processing associated with the current submission is available at https://github.com/dtm2451/EndometrialDeconvolution and https://github.com/dtm2451/EndometriosisDrugRepurposing, and code for the computational drug repurposing pipeline associated with the current submission is available at https://github.com/Bin-Chen-Lab/HCC_NEN.
• Any additional information required to reanalyze the data reported in this study is available from the lead contact upon request.

## Acknowledgments

The authors acknowledge the use of resources developed and supported by the UCSF Bakar Computational Health Sciences Institute Information Commons team, and thank members of this team for technical support. The authors also thank the Center for Data-driven Insights and Innovation at UC Health, and the members of their team for technical support.

NIH Eunice Kennedy Shriver National Institute of Child Health and Human Development (10.13039/100000071NICHD) P01 HD106414 (T.T.O., X.T., A.B., J.C.I., B.G., D.K.S., L.C.G., S.L.M., and M.S.).

NIH 10.13039/100000071NICHD P50 HD055764 (A.B., J.C.I., L.C.G., and M.S.).

NIH 10.13039/100000071NICHD R00 HD093858 (S.L.M.).

March of Dimes Prematurity Research Center at 10.13039/100008069UCSF (T.T.O. and M.S.).

March of Dimes Prematurity Research Center at 10.13039/100005492Stanford University (B.G. and D.K.S.).

Stanford Maternal and Child Health Research Institute (B.G. and D.K.S.).

Emory Integrated Genomics Core (EIGC) (RRID:SCR_023529), which is subsidized by the 10.13039/100007623Emory University School of Medicine and is one of the Emory Integrated Core Facilities. 10.13039/100016220Georgia Clinical and Translational Science Alliance of the 10.13039/100000002National Institutes of Health under award number UL1TR002378.

The content is solely the responsibility of the authors and does not necessarily reflect the official views of the National Institutes of Health.

## Author contributions

Conceptualization, T.T.O., X.T., L.C.G., S.L.M., and M.S.; methodology, T.T.O., X.T., E.A., A.G., A.L., and S.L.M.; investigation, T.T.O., X.T., E.A., A.G., A.L., and S.L.M.; visualization, T.T.O., X.T., F.A., and S.L.M.; funding acquisition, B.G., D.K.S., L.C.G., S.L.M., and M.S.; supervision, B.G., D.K.S., L.C.G., S.L.M., and M.S.; writing – original draft, T.T.O., X.T., E.A., A.G., F.A., and S.L.M.; writing – review and editing, T.T.O., X.T., E.A., A.G., A.L., F.A., D.G.B., J.E., M.D., J.C.I., B.G., D.K.S., L.C.G., S.L.M., and M.S.

## Declaration of interests

A.B., D.G.B., T.T.O., L.C.G., and M.S. have a patent related to this work. L.C.G. is a paid consultant to Gensyta Pharma, Celmatix, and NextGen Jane. M.S. is an advisor to Wellcome Leap. The remaining authors declare no competing interests.

## STAR★Methods

### Key resources table

**Table:** 

REAGENT or RESOURCE	SOURCE	IDENTIFIER
**Deposited data**		

A Transcriptomics-Based Computational Drug Repositioning Pipeline Identifies Simvastatin And Primaquine As Therapeutics For Endometriosis	This manuscript	Gene Expression Omnibus (GEO), GEO: GSE296883

**Experimental models: Organisms/strains**		

Sprague-Dawley rats	Charles River (Wilmington, MA; Raleigh NC facility)	Crl:CD(SD); RRID: RGD_734476

**Software and algorithms**		

Endometrial Deconvolution code	Bunis et al.^42^	https://github.com/dtm2451/EndometrialDeconvolution
Endometriosis Drug Repurposing – Differential Gene Expression code	Bunis et al.^42^	https://github.com/dtm2451/EndometriosisDrugRepurposing
Drug repurposing pipeline	Chen et al.^20^	https://github.com/Bin-Chen-Lab/HCC_NEN

**Other**		

University of California Health Data Warehouse (UCHDW)	Center for Data-driven Insights and Innovation (CDI2)	N/A

### Experimental model and study participant details

#### Animal study cohort

A total of 18 adult virgin female Sprague-Dawley rats were used in the animal study, each weighing between 175 and 225 grams at the onset of the study, and were sourced from Charles River Laboratory (Wilmington, MA; Raleigh, NC facility). Our study exclusively examined female rats because the disease modeled is only relevant in females. The animals were housed individually in standard rodent cages with access to water and chow *ad libitum*. They were kept under controlled conditions with a 12-hour light/dark cycle (lights on at 07:00), and the room temperature was maintained at ∼22°C. Estrous cycle stages were monitored and documented daily 2 hours after lights on via vaginal lavage. The animal study and procedures were approved by the Emory University Institutional Animal Care and Use Committee (IACUC) as protocol #2021000201. All laboratory animal experimentation adhered to the National Institutes of Health (NIH) Guide for the Care and Use of Laboratory Animals. Animal experiments are reported in accordance with Animal Research: Reporting of *In Vivo* Experiments (ARRIVE) guidelines.

#### Human participants - electronic medical records cohort

A HIPAA (Health Insurance Portability and Accountability Act)-compliant limited dataset of electronic medical records (January 1, 2012 - May 30, 2025) representing patients across six University of California health systems (UC Davis, UC Irvine, UCLA, UC Riverside, UC San Diego, UCSF) was accessed via the University of California Data Discovery Platform (UCDDP) and analyzed in August 2025. Patients who were of female sex were included. The electronic medical records analysis was approved by the University of California, San Francisco Institutional Review Board (IRB) as protocol #22-37954.

### Method details

#### Computational drug repositioning

In previous work,^23^ our computational drug repositioning pipeline was applied to bulk transcriptomic data, which consisted of 105 samples from eutopic endometrial tissues of women with and without endometriosis. On the drug side, the Connectivity Map (CMap) dataset was used to obtain gene expression profiles from cell lines treated with existing small-molecule drugs. Through this approach, potential therapeutics were identified based on reversal of endometriosis gene expression signatures that were unstratified as well as stratified by ASRM disease stage (ASRM stages I/II and III/IV) and menstrual cycle phase (proliferative, early secretory, and mid-secretory phases) as described in Bunis et al.^42^ As a proof of principle, the top therapeutic candidate fenoprofen, an NSAID infrequently prescribed for endometriosis, was validated in an animal model of endometriosis.

In this study, among the 299 unique drug candidates that were previously identified, those among the top-ranked (i.e., among the top 10%) were evaluated for further consideration, which included primaquine and simvastatin. Factors such as safety profiles, availability (i.e., on the World Health Organization list of essential medicines^64^), and ease of administration (e.g., does not require intravenous administration) were taken into consideration.

#### Animal studies

The primary aim of the animal study was to evaluate the effects of simvastatin and primaquine on pain-associated behavior in a rat model of endometriosis. Endometriosis-associated hyperalgesia has been assessed across preclinical rodent models using behavioral endpoints, including vaginal distension-evoked escape responses in rat models and evoked mechanical sensitivity in mouse models.^62^^,^^65^^,^^66^ A sample size of six rats per group was selected based on prior studies using this rat endometriosis model and vaginal distension-evoked escape responses.^62^ The within-subject design allowed each animal to serve as its own baseline control across baseline, post-endo, and post-treatment phases, where post-endo refers to the period after endometriosis induction but before treatment. At baseline, estrous cycles were monitored and nociceptive behavior was characterized in all animals. Endometriosis was then surgically induced, and rats received simvastatin, primaquine, or vehicle (negative control) for 4 weeks. Behavioral assessments of vaginal nociception were conducted after induction and again after treatment to determine treatment effects. All training and testing occurred 3–8 hours after lights-on, three times per week on non-consecutive days.

#### Endometriosis induction

Endometriosis was surgically induced in rats as described by Vernon and Wilson.^52^ Animals were anesthetized with a combination of ketamine (73 mg/kg) and xylazine (8.8 mg/kg), and a midline abdominal incision was made. A 1 cm segment of the left uterine horn with surrounding fat was excised and transferred to sterile saline. This tissue was cut into four 2x2 mm fragments and sutured onto the mesenteric arteries supplying the small intestine. After the surgical procedure, the incision was closed, and the rats were closely monitored during recovery. The operation had no complications and estrous cycles resumed within a few days.

#### Drug administration

After endometriosis-like lesions were established, rats were randomly assigned (*n* = 6 per group) to receive simvastatin (40 mg/kg/day), primaquine (40 mg/kg/day), or vehicle by oral gavage once daily for 4 weeks. The 40 mg/kg/day dose for both agents was selected from prior rodent studies showing activity and tolerability in inflammatory and nociceptive models.^35^^,^^67^^,^^68^^,^^69^^,^^70^^,^^71^ Vehicle animals received an equal volume of dosing vehicle without active drug. Behavioral testing and outcome analysis were performed by investigators blinded to treatment assignment.

#### Assessment of vaginal nociception

Vaginal nociception was assessed by quantifying escape responses to graded vaginal distension, as previously described for this rat model of endometriosis-associated vaginal hyperalgesia.^62^^,^^66^ Briefly, rats were exposed to vaginal distension via inflation of a latex balloon inserted into the vaginal canal. The primary behavioral endpoint was the percentage of successful escape responses, with animals trained to break a light beam by extending their heads into a tube in response to distension. This procedure was repeated at eight different balloon volumes, three times each in random order, to characterize volume-dependent changes in nociceptive escape behavior.

#### Behavioral testing apparatus

The testing apparatus was a rectangular Plexiglas chamber equipped with a grid floor, which restricted the rat from turning around. A hollow tube containing light-emitting diodes and a photosensor extended from the front of the chamber. When the rat extended its head, it interrupted the light beam, resulting in the cessation of the stimulus and the provision of peanut butter as a reward by the experimenters. This action, referred to as an escape response, was used to measure behavioral responses to the vaginal distension.

An opening in the rear of the chamber allowed the balloon-tipped catheter to be connected to the computer-controlled and automated stimulus-delivery device. The latex balloon (10 mm long, 1.5 mm wide when uninflated) was lubricated with K-Y® jelly and inserted into the vaginal canal of the rat before each session. It was then inflated to different volumes through the computer-controlled pump to induce vaginal distension at 60-second intervals, with the pressure produced by each volume monitored via a small-volume Cobe pressure transducer and the escape response to each volume recorded.

#### Behavioral training

Training began by acclimating the rats to the testing chamber for 10 minutes daily for 3–4 days, during which small amounts of peanut butter were provided on a wooden stick. The rats were then trained by using tail pinches with padded forceps, which prompted them to extend their head into the tube to break the light beam. Once the light beam was broken, the tail pinch was released to reinforce the “escape response,” and the rats were rewarded with peanut butter if they successfully interrupted the light beam to escape the tail pinch. This process was repeated for 10 tail pinches at 1-minute intervals over 3 training sessions each week on non-consecutive days. Training was completed (>80% escape behavior) in 4–8 sessions.

The rats were next trained to make identical escape responses to deflate vaginal distension stimuli. These sessions were run 3 times/week on non-consecutive days for a total of 3–5 sessions. Ten large distension volumes (0.80 ml – 1.0 ml, inflation rate 1 ml/s) were delivered for a maximum of 15 s at 1-min intervals. All rats showed some behavioral response to these stimuli, which allowed the experimenter to use deflation of the vaginal balloon to shape the rat’s escape responses. All rats learned the escape response within 2–4 sessions. Once trained, testing sessions began.

#### Experimental procedure

Behavioral testing was conducted across three phases: baseline (prior to endometriosis induction), post-endo (after endometriosis induction but before treatment), and post-treatment (after drug/control administration). In each phase, rats underwent a series of 24 trials (8 different balloon inflation volumes delivered 3 times each). Each trial involved inflating the balloon at a constant rate of 1 mL/s and maintaining the inflation for up to 15 seconds unless an escape response occurred. The balloon was then deflated at 0.5 mL/s if an escape response occurred or if 15 seconds elapsed. The percentage of successful escape responses was recorded for analysis.

#### RNA Seq

##### Experimental procedure

RNAs were extracted using miRNeasy mini Kit (Qiagen), and 1.5 μg of total RNA was used for library construction. The libraries for RNA sequencing were prepared using the Illumina Stranded Total RNA Prep with Ribo-Zero^TM^ Plus kit. We sequenced libraries using Novaseq X Plus (Illumina), paired-end, 100 reads up to 50M paired reads per sample.

#### Electronic medical records

The number of individuals who were ever prescribed primaquine or simvastatin was assessed, and investigation of the compound of interest (COI) proceeded if there was sufficient power for downstream analysis. Our analysis compared endometriosis diagnoses among individuals with a prescription for the COI to a propensity-score–matched cohort without any such prescription. Conditions identified by using International Classification of Diseases, Ninth Revision, Clinical Modification (ICD-9-CM), and International Statistical Classification of Diseases, Tenth Revision, Clinical Modification (ICD-10-CM), diagnosis codes include endometriosis (617.X, N80.X) and high cholesterol/hyperlipidemia (272.0X, 277.1X, 277.2X, 277.3X, 277.4X, E78.0X, E78.1X, E78.2X, E78.3X, E78.4X, E78.5X).

Among patients with a prescription for the COI, they were included only if their first COI prescription occurred at age 40 or younger and at least five years had passed since that first COI prescription date. Individuals who were younger than 12 years old, missing information for sex, race, ethnicity, UC healthcare site, or number of visits, or had an endometriosis diagnosis prior to their first COI prescription were excluded.

### Quantification and statistical analysis

#### Statistical analysis – animal studies

No animals were excluded from the behavioral analyses. For each rat group (VEH: with endo surgery, vehicle treatment (vehicle control) (*n* = 6); SIMVA: with endo surgery, treated with simvastatin (*n* = 6); PRIMA: with endo surgery, treated with primaquine (*n* = 6)); and the historical comparison groups from previous work^23^ (CNS: control, no endo surgery; IBU: ibuprofen; FEN: fenoprofen), a non-parametric test was performed to compare within groups, the escape responses of the 3 testing periods: baseline (24 data points/rat), post-endo surgery (64 data points/rat), and post-treatment (32 data points/rat). Mann Whitney U (MWU) tests were performed to compare the escape responses of (a) the baseline period and the post-endo period, (b) the post-endo period and the post-treatment period, and (c) the baseline period and the post-treatment period at each volume of water (0.01, 0.15, 0.30, 0.40, 0.55, 0.70, 0.80, and 0.90 mL) delivered to the balloon placed within the mid-vaginal canal of the rats. Moreover, Mann Whitney U (MWU) tests were performed to compare between groups, the escape responses of all pairwise group comparisons for each of the three condition periods (the baseline period, the post-endo period, and post-treatment period) at each volume of water (0.01, 0.15, 0.30, 0.40, 0.55, 0.70, 0.80, and 0.90 mL) delivered to the balloon placed within the mid-vaginal canal of the rats. Escape responses reflect the percentage of times rats extended their head into the tube to interrupt the light beam to terminate the stimulus (balloon deflates). A significance threshold of 0.05 was applied to Bonferroni–corrected *p*-values. Data are presented as median and interquartile range (IQR).

#### Statistical analysis–RNAseq

Adapter sequences in the raw read files were trimmed using cutadapt (v4.9) with an error rate of 0.1, minimum overlap of 3 bps, minimum 3′ base quality score of 25, and minimum length after trimming of 55 bps. We also set to remove poly-A tails and poly-G with lengths over 20 bps. The trimmed reads were mapped to *Rattus* *norvegicus* reference genome GRCr8 with exon and splice site information using Hisat2 (v2.2.0). The SAM files were converted to BAM files and sorted using samtools (v1.21). The gene counts were summarized by featureCounts (v2.0.6).

For the dimensionality reduction, gene counts were log2 transformed and the top 3000 most variable genes based on their standard deviation were used to perform principal component analysis (PCA).

The differential gene expression analysis comparing treatment groups was performed using the edgeR package (v4.4.1) in R 4.4.2. We first excluded genes that were uncharacterized from further analysis. Low-expressed genes were dropped using the filterByExpr() function based on the treatment factors. After filtering, we recalculated the library sizes. To account for sample-specific effects, we applied the trimmed mean of M-values (TMM) normalization. We then estimated the dispersions for all genes and fitted a negative binomial generalized log-linear model to the gene counts. This model included an interaction term between the sample source and treatment, with the RNA Integrity Number (RIN) included as a covariate. The differential expression of genes between treatment groups within each sample source was tested with genewise quasi F-tests for the coefficient contrasts of the model. P-values were subjected to the Benjamini-Hochberg (BH) method to control the false positive rate. A Benjamini-Hochberg (BH)-adjusted *p*-value threshold of 0.05 was used to determine significance. Gene set enrichment analysis and over-representation analysis were performed using the clusterProfiler package (version 4.14.4). Pearson correlation coefficients were calculated to assess gene expression correlations between treatment groups. The overlap between human disease signatures and rat differentially expressed genes was evaluated using the hypergeometric test.

#### Statistical analysis - electronic medical records

Propensity score matching was conducted with the MatchIt package (R, v4.7.2) using nearest-neighbor matching at a 1:4 treated-to-control ratio. Propensity scores were estimated via logistic regression of treatment on baseline demographics (age, race, ethnicity), UC healthcare site, number of visits, and medication indication. Covariate balance was assessed with balance plots of the standardized mean differences for covariates before and after propensity-score matching.

The matching procedure was repeated 10 times to address uncertainty from tied matches. Each iteration included all treated patients and a re-sampled, propensity-matched control subset. For each iteration, the relative risk of endometriosis diagnosis (with 95% confidence intervals) was estimated and results summarized across iterations to evaluate robustness. Multiple hypothesis testing was controlled using the Benjamini-Hochberg procedure with an adjusted significance threshold of 0.05.

## Supplemental information

Document S1. Figures S1–S6

Table S1. Drug candidates identified via computational drug repositioning pipeline using unstratified and stratified bulk transcriptomic signatures, related to Figure 1

Table S2. Pairwise Mann-Whitney U comparisons, with Bonferroni correction, of the six treatment groups by volume, related to Figure 3

Table S3. Differentially expressed genes in lesion samples in FEN treatment group, related to Figure 4

Table S4. Differentially expressed genes in lesion samples in IBU treatment group, related to Figure 4

Table S5. Differentially expressed genes in lesion samples in PRIMA treatment group, related to Figure 4

Table S6. Differentially expressed genes in lesion samples in SIMVA treatment group, related to Figure 4

Table S7. Differentially expressed genes in uterus samples in VEH treatment group, related to Figure 4

Table S8. Differentially expressed genes in uterus samples in FEN treatment group, related to Figure 4

Table S9. Differentially expressed genes in uterus samples in IBU treatment group, related to Figure 4

Table S10. Differentially expressed genes in uterus samples in PRIMA treatment group, related to Figure 4

Table S11. Differentially expressed genes in uterus samples in SIMVA treatment group, related to Figure 4
