# Supplementary material for: A transcriptomics-based computational drug repurposing pipeline identifies simvastatin and primaquine as therapeutics for endometriosis
Source: iScience. 2026 Aug 21;29(9):117153. doi: 10.1016/j.isci.2026.117153 (PMC13524725; doi:10.1016/j.isci.2026.117153)

**iScience, Volume *29***

## Supplemental information

# A transcriptomics-based computational drug repurposing pipeline identifies simvastatin and primaquine as therapeutics for endometriosis

**Tomiko T. Oskotsky, Xinyu Tang, Erin Arthurs, Arpita Govil, Ferheen Abbasi, Arohee Bhoja, Daniel G. Bunis, Abby Lau, Jakob Einhaus, Maïgane Diop, Juan C. Irwin, Brice Gaudilliere, David K. Stevenson, Linda C. Giudice, Stacy L. McAllister, and Marina Sirota**

# SUPPLEMENTARY FIGURES

| Volume (mL) | Baseline period (BL) median | BL IQR | Post-Endo period (PE) median | PE IQR | Post-Treatment period (PT) median | PT IQR | BL vs PE period MWU test Bonferroni-adjusted p-values | PE vs PT period MWU test Bonferroni-adjusted p-values | BL vs PT period MWU test Bonferroni-adjusted p-values |
|---|---|---|---|---|---|---|---|---|---|
| **CONTROL NO SURGERY (Negative control)** | | | | | | | | | |
| 0.01 | 0 | (0-0) | 0 | (0-0) | 0 | (0-0) | 1 | 1 | 1 |
| 0.15 | 0 | (0-0) | 0 | (0-0) | 0 | (0-0) | 1 | 1 | 1 |
| 0.30 | 0 | (0-0) | 0 | (0-33.3) | 0 | (0-33.3) | 1 | 1 | 1 |
| 0.40 | 33.3 | (0-33.3) | 33.3 | (0-33.3) | 33.3 | (0-33.3) | 1 | 1 | 1 |
| 0.55 | 33.3 | (33.3-33.3) | 33.3 | (33.3-66.6) | 33.3 | (33.3-66.6) | 1 | 1 | 1 |
| 0.70 | 66.6 | (33.3-66.6) | 66.6 | (66.6-66.6) | 66.6 | (66.6-66.6) | 1 | 1 | 1 |
| 0.80 | 100 | (66.6-100) | 100 | (66.6-100) | 100 | (66.6-100) | 1 | 1 | 1 |
| 0.90 | 100 | (100-100) | 100 | (100-100) | 100 | (100-100) | 1 | 1 | 1 |
| **VEHICLE (Negative control)** | | | | | | | | | |
| 0.01 | 0 | (0-0) | 0 | (0-0) | 0 | (0-0) | 1 | 1 | 1 |
| 0.15 | 0 | (0-0) | 33.3 | (33.3-66.6) | 33.3 | (33.3-33.3) | 1.7E-05 **** | 1 | 1.1E-05 **** |
| 0.30 | 0 | (0-33.3) | 66.6 | (33.3-66.6) | 66.6 | (33.3-66.6) | 9.8E-06 **** | 1 | 7.0E-06 **** |
| 0.40 | 33.3 | (0-33.3) | 66.6 | (66.6-100) | 66.6 | (66.6-66.6) | 4.1E-08 **** | 1 | 1.1E-06 **** |
| 0.55 | 33.3 | (33.3-41.6) | 100 | (66.6-100) | 100 | (75.0-100) | 1.7E-11 **** | 1 | 4.8E-09 **** |
| 0.70 | 66.6 | (58.3-75.0) | 100 | (100-100) | 100 | (100-100) | 4.3E-06 **** | 1 | 2.3E-05 **** |
| 0.80 | 100 | (66.6-100) | 100 | (100-100) | 100 | (100-100) | 6.1E-05 **** | 1 | 0.02 * |
| 0.90 | 100 | (100-100) | 100 | (100-100) | 100 | (100-100) | 1 | 1 | 1 |
| **IBUPROFEN (Positive control)** | | | | | | | | | |
| 0.01 | 0 | (0-0) | 0 | (0-0) | 0 | (0-0) | 1 | 1 | 1 |
| 0.15 | 0 | (0-0) | 16.65 | (0-33.3) | 0 | (0-0) | 3.5E-03 ** | 2.3E-03 ** | 1 |
| 0.30 | 0 | (0-0) | 33.3 | (0-66.6) | 0 | (0-0) | 2.0E-03 ** | 9.7E-04 ** | 1 |
| 0.40 | 0 | (0-25) | 66.6 | (33.3-66.6) | 0 | (0-33.3) | 1.5E-05 **** | 2.9E-06 **** | 1 |
| 0.55 | 33.3 | (8.3-33.3) | 83.3 | (66.6-100) | 0 | (0-33.3) | 2.5E-07 **** | 3.4E-08 **** | 1 |
| 0.70 | 66.6 | (66.6-66.6) | 100 | (100-100) | 66.6 | (33.3-66.6) | 5.8E-05 **** | 5.1E-06 **** | 1 |
| 0.80 | 100 | (66.6-100) | 100 | (100-100) | 100 | (66.6-100) | 3.9E-03 ** | 1.8E-03 ** | 1 |
| 0.90 | 100 | (74.9-100) | 100 | (100-100) | 100 | (100-100) | 0.12 | 0.8 | 1 |
| **FENOPROFEN** | | | | | | | | | |
| 0.01 | 0 | (0-0) | 0 | (0-0) | 0 | (0-0) | 1 | 1 | 1 |
| 0.15 | 0 | (0-0) | 33.3 | (0-66.6) | 0 | (0-0) | 2.7E-04 *** | 2.3E-03 ** | 0.61 |
| 0.30 | 0 | (0-25) | 33.3 | (33.3-66.6) | 0 | (0-33.3) | 2.0E-05 **** | 3.0E-06 **** | 1 |
| 0.40 | 0 | (0-33.3) | 66.6 | (33.3-91.7) | 16.65 | (0-33.3) | 4.0E-06 **** | 1.8E-05 **** | 1 |
| 0.55 | 33.3 | (8.3-33.3) | 83.3 | (66.6-100) | 33.3 | (33.3-66.6) | 2.7E-07 **** | 7.9E-07 **** | 0.16 |
| 0.70 | 66.6 | (33.3-66.6) | 100 | (100-100) | 66.6 | (66.6-100) | 1.5E-03 ** | 4.4E-02 * | 0.97 |
| 0.80 | 66.6 | (66.6-100) | 100 | (100-100) | 100 | (66.6-100) | 4.8E-05 **** | 5.8E-03 * | 1 |
| 0.90 | 100 | (100-100) | 100 | (100-100) | 100 | (100-100) | 0.34 | 0.8 | 1 |
| **PRIMAQUINE** | | | | | | | | | |
| 0.01 | 0 | (0-0) | | (0-0) | 0 | (0-0) | 1 | 1 | 1 |
| 0.15 | 0 | (0-0) | 33.3 | (0-66.6) | 0 | (0-33.3) | 3.7E-06 **** | 1.4E-03 ** | 0.24 |
| 0.30 | 0 | (0-0) | 50 | (33.3-66.6) | 33.3 | (0-33.3) | 1.2E-07 **** | 1.7E-02 * | 2.6E-03 ** |
| 0.40 | 33.3 | (0-33.3) | 66.6 | (41.6-100) | 50 | (33.3-66.6) | 4.5E-09 **** | 3.3E-02 * | 1.6E-05 **** |
| 0.55 | 33.3 | (33.3-41.6) | 100 | (66.6-100) | 66.6 | (33.3-100) | 3.4E-10 **** | 1.0E-03 ** | 0.03 * |
| 0.70 | 50 | (33.3-66.6) | 100 | (100-100) | 66.6 | (66.6-100) | 6.5E-09 **** | 5.8E-03 * | 9.8E-04 ** |
| 0.80 | 66.6 | (66.6-100) | 100 | (100-100) | 100 | (66.6-100) | 6.2E-03 * | 9.3E-02 | 1 |
| 0.90 | 100 | (100-100) | 100 | (100-100) | 100 | (100-100) | 0.47 | 1 | 1 |
| **SIMVASTATIN** | | | | | | | | | |
| 0.01 | 0 | (0-0) | 0 | (0-0) | 0 | (0-0) | 1 | 1 | 1 |
| 0.15 | 0 | (0-0) | 33.3 | (33.3-66.6) | 0 | (0-0) | 6.8E-08 **** | 1.7E-09 **** | 1 |
| 0.30 | 0 | (0-8.3) | 66.6 | (41.6-66.6) | 0 | (0-25.0) | 2.0E-11 **** | 1.0E-12 **** | 1 |
| 0.40 | 33.3 | (0-33.3) | 66.6 | (66.6-100) | 33.3 | (33.3-33.3) | 6.0E-11 **** | 3.9E-08 **** | 0.08 |
| 0.55 | 33.3 | (33.3-66.6) | 100 | (66.6-100) | 33.3 | (33.3-66.6) | 5.4E-09 **** | 1.1E-07 **** | 1 |
| 0.70 | 66.6 | (33.3-66.6) | 100 | (100-100) | 66.6 | (41.6-100) | 5.0E-07 **** | 2.3E-04 *** | 1 |
| 0.80 | 83.3 | (66.6-100) | 100 | (100-100) | 83.3 | (66.6-100) | 9.0E-05 **** | 1.5E-05 **** | 1 |
| 0.90 | 100 | (100-100) | 100 | (100-100) | 100 | (100-100) | 1 | 1 | 1 |

**Fig. S1: Endometriosis animal model escape responses.** Median escape response with interquartile range (IQR) for each delivered volume (0.01, 0.15,0.30, 0.40, 0.55, 0.70, 0.80, and 0.90 mL) during the baseline, post-endo surgery, and post-treatment periods, with Bonferroni-corrected p-values from Mann-Whitney U test for baseline period vs post-endo surgery period, post-endo surgery vs post-treatment period, and baseline period vs post-treatment period (* denotes <0.05, ** denotes <0.005, *** denotes <0.0005, and **** denotes <0.0001). Endo rats (n=6/group) vaginal nociception assessed. Treated with vehicle (negative control), primaquine, simvastatin, fenoprofen, or ibuprofen (positive control). Note: Fenoprofen and ibuprofen data are from prior work[23]

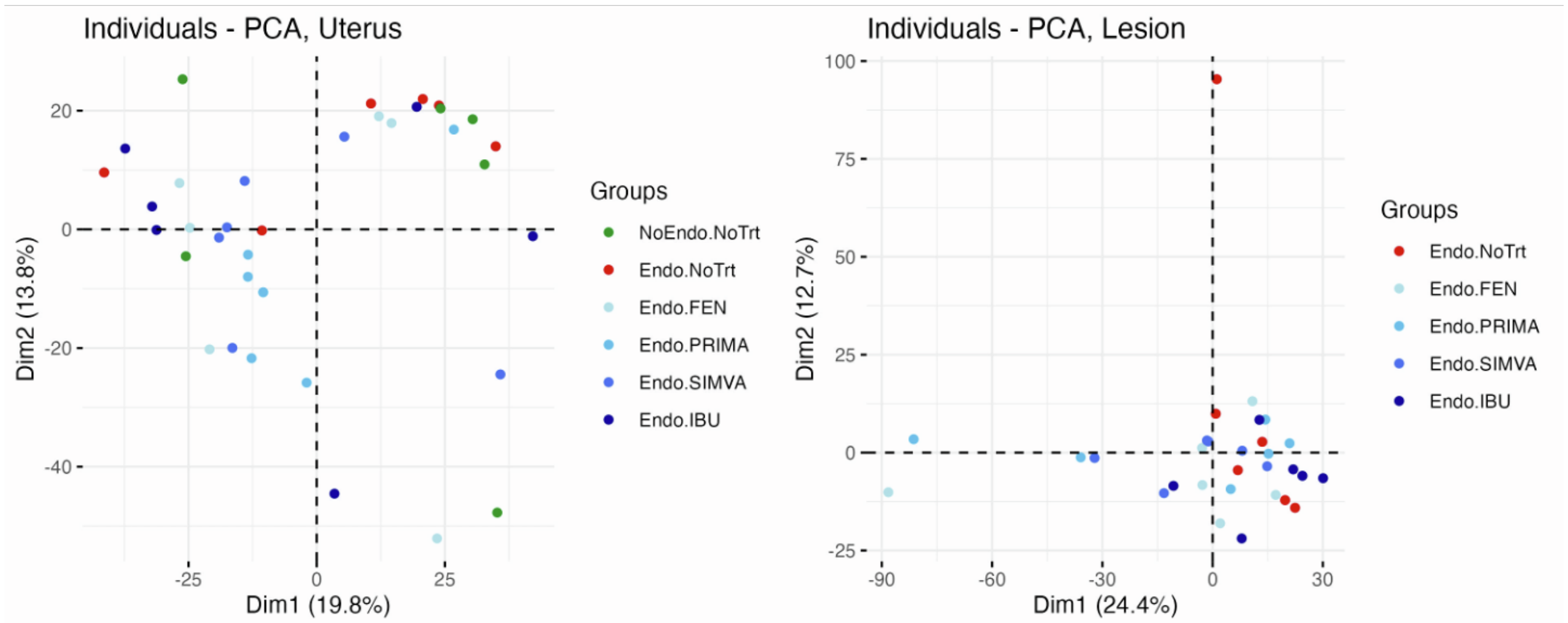


**Fig. S2. Principal component analysis (PCA) for each sample source** -- uterus (left) and lesion (right). Each dot represents a sample.

| Lesion | FEN | PRIMA | SIMVA | IBU |
|---|---|---|---|---|
| Total Genes | 17504 | 17504 | 17504 | 17504 |
| DEG P<0.05 | 1621 | 1496 | 974 | 1073 |
| DEG FDR<0.05 | 190 | 233 | 105 | 116 |

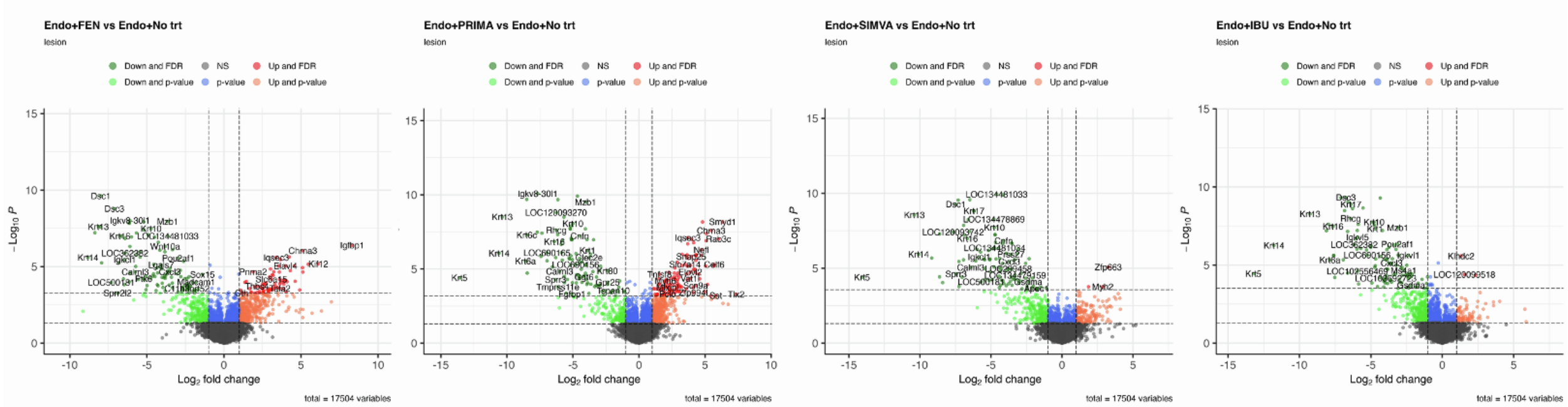


**Fig. S3. Volcano plots of individual differentially expressed genes (DEGs) in lesion samples by treatment group**. The x-axis reflects the negative (-) $\log_{10}$ of the Benjamini-Hochberg (BH)-adjusted p-value, and the y-axis reflects the $\log_2$ fold change ($\log_2FC$). Differentially expressed genes (DEGs) with False Discovery Rate (FDR) < 0.05 and $|\log_2FC| > 1$ are shown in dark red (upregulated) or dark green (downregulated). Genes with nominal p-value < 0.05 and $|\log_2FC| > 1$, but not meeting the FDR threshold, are shown in light red or light green.

| Uterus | Endo | FEN | PRIMA | SIMVA | IBU |
|---|---|---|---|---|---|
| Total Genes | 17504 | 17504 | 17504 | 17504 | 17504 |
| DEG P<0.05 | 762 | 1566 | 3481 | 1983 | 1420 |
| DEG FDR<0.05 | 1 | 19 | 168 | 2 | 0 |

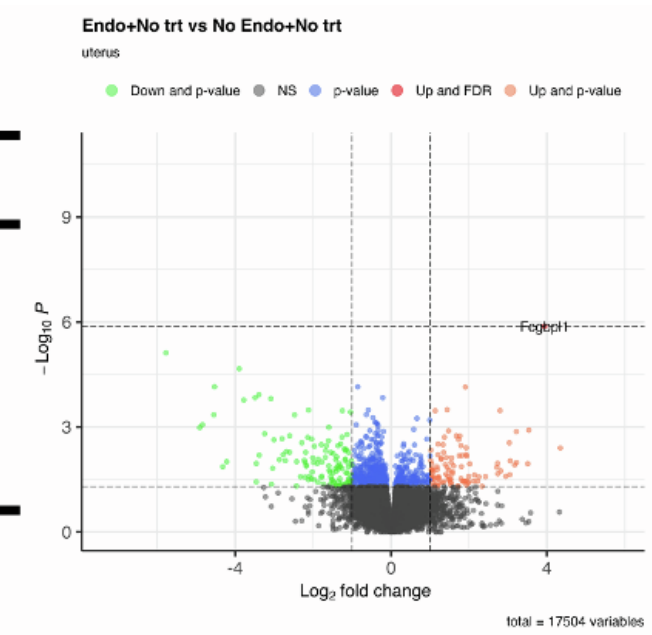


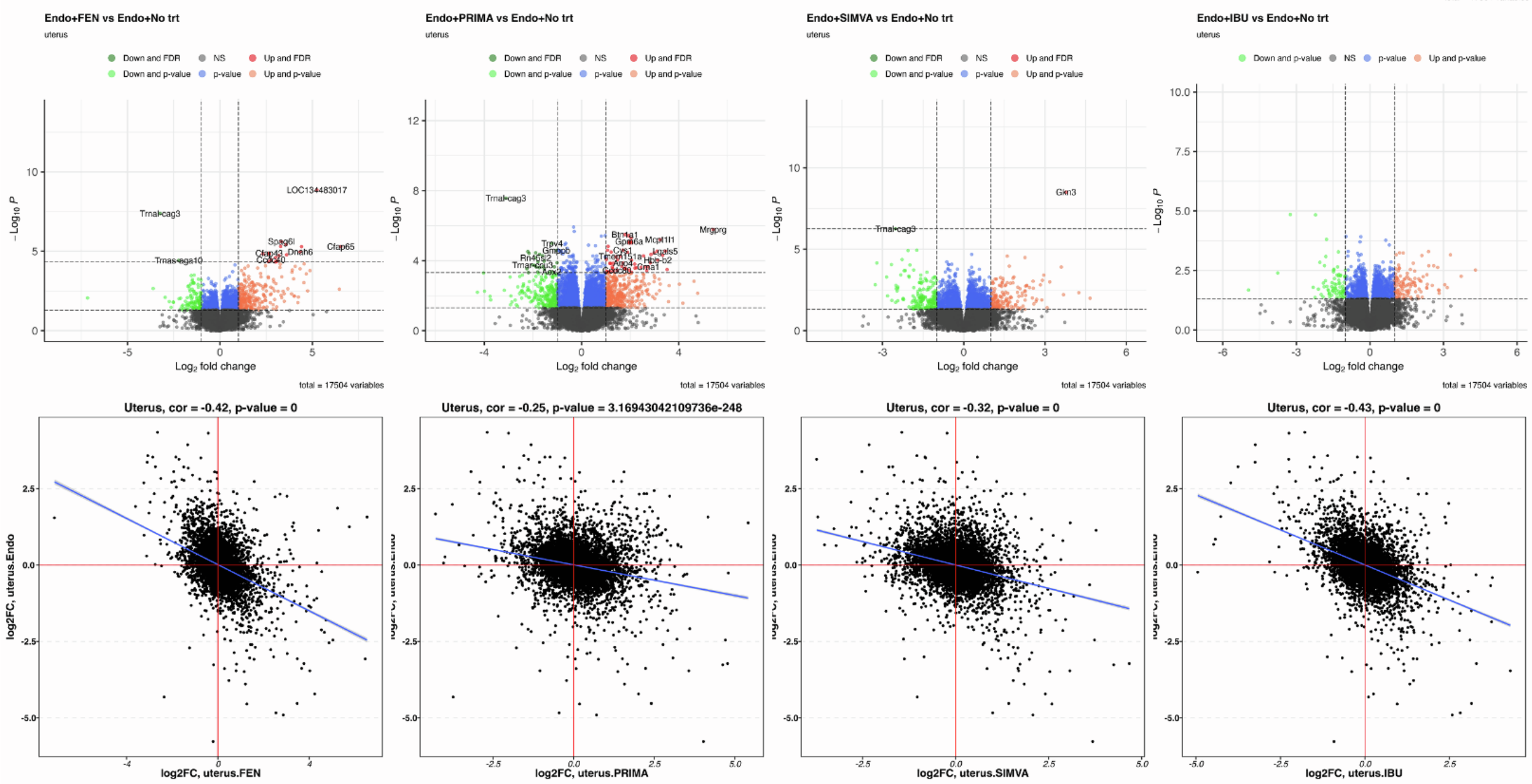


**Fig. S4. Volcano plots of individual differentially expressed genes (DEGs) in uterus samples by treatment group.** The x-axis reflects the negative (-) $\log_{10}$ of the Benjamini-Hochberg (BH)-adjusted p-value, and the y-axis reflects the $\log_2$ fold change. Differentially expressed genes (DEGs) with FDR < 0.05 and $|\log_2 FC| > 1$ are shown in dark red (upregulated) or dark green (downregulated). Genes with nominal p-value < 0.05 and $|\log_2 FC| > 1$, but not meeting the FDR threshold, are shown in light red or light green.

**Fig. S5. Overview of the Analysis for Simvastatin in Electronic Medical Records**

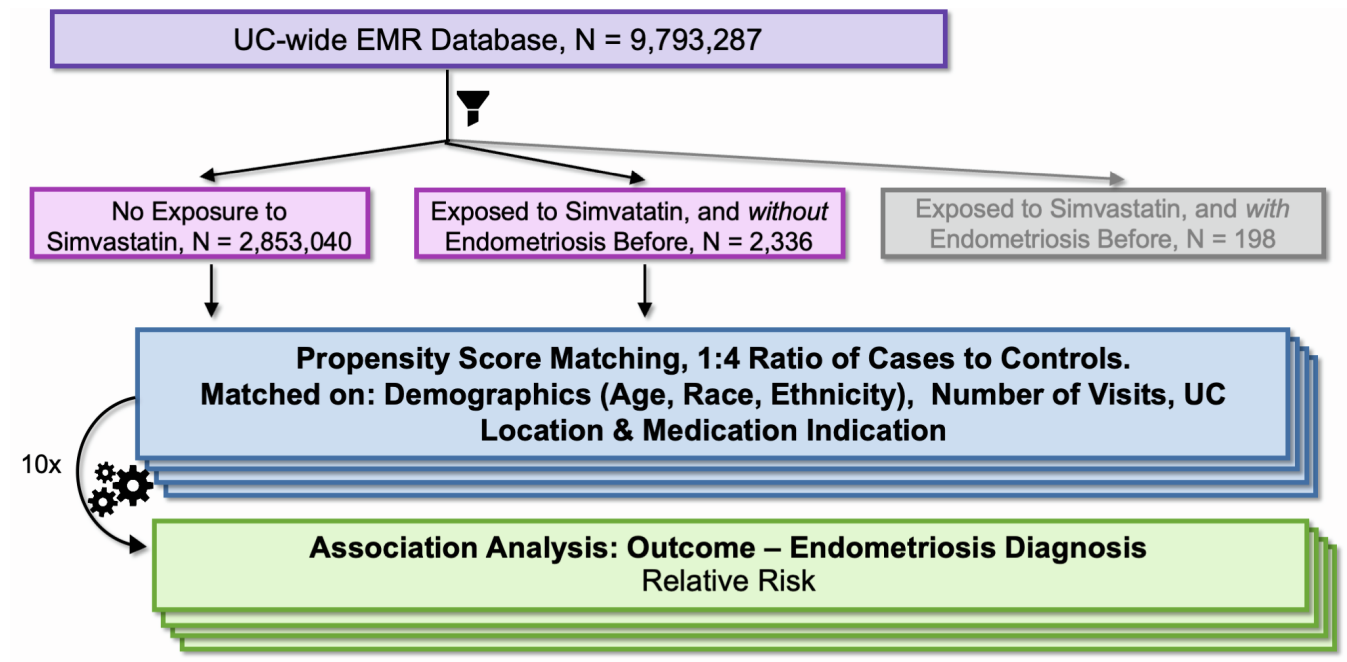


**Fig. S6. Balance plot of the absolute standardized mean difference (SMD) of covariates before PS-matching (unadjusted, in pink) and after PS-matching (adjusted, in blue)**, with an absolute SMD of less than (0.1, reflected by the red-dashed line) indicative of adequate balance between groups.

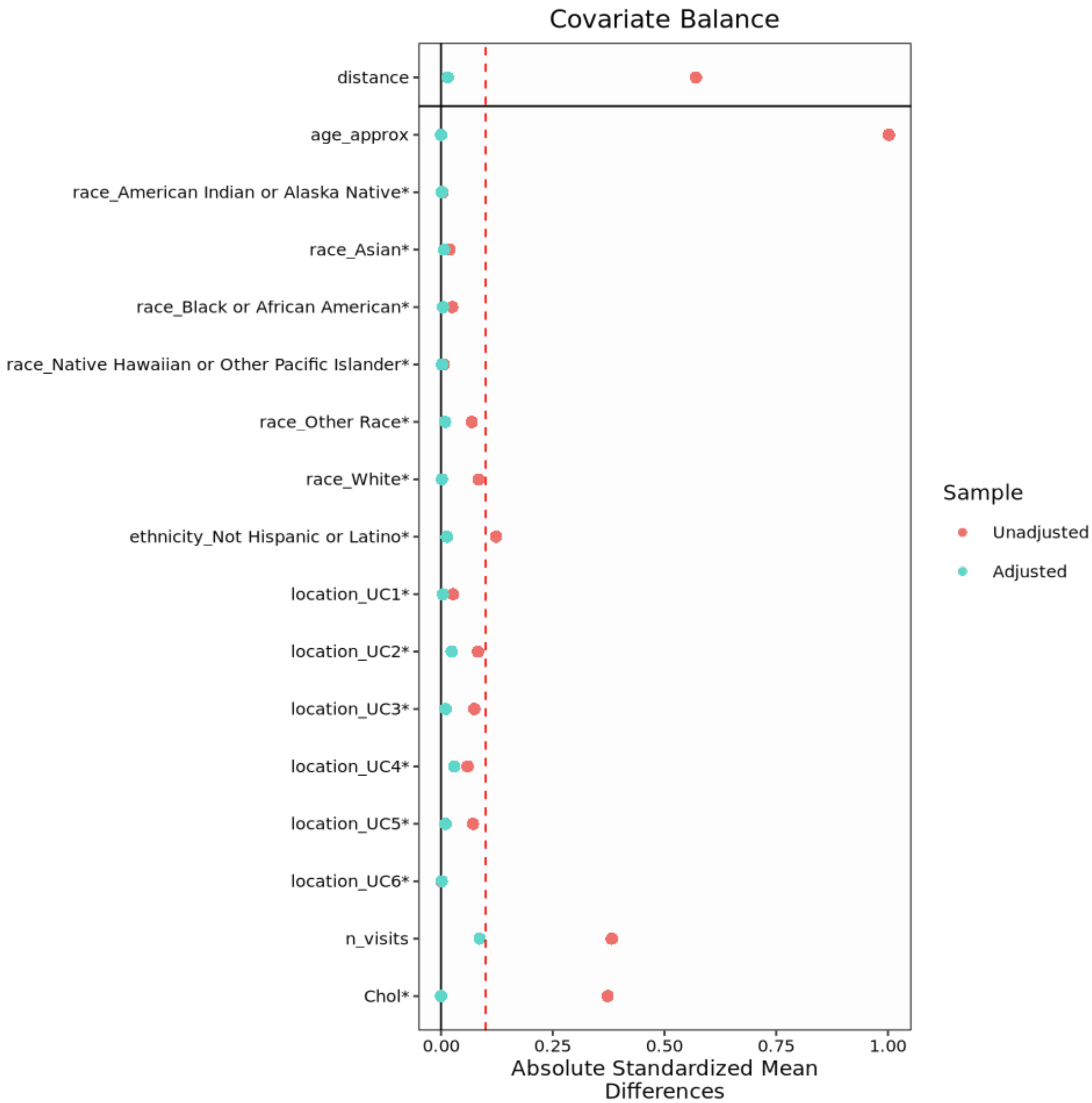

Supplement: Supplementary file 1 [file mmc1.pdf]
